# Acquisition of Human-Type Receptor Binding Specificity by New H5N1 Influenza Virus Sublineages during Their Emergence in Birds in Egypt

**DOI:** 10.1371/journal.ppat.1002068

**Published:** 2011-05-26

**Authors:** Yohei Watanabe, Madiha S. Ibrahim, Hany F. Ellakany, Norihito Kawashita, Rika Mizuike, Hiroaki Hiramatsu, Nogluk Sriwilaijaroen, Tatsuya Takagi, Yasuo Suzuki, Kazuyoshi Ikuta

**Affiliations:** 1 Department of Virology, Research Institute for Microbial Diseases (BIKEN), Osaka University, Osaka, Japan; 2 Department of Microbiology, Faculty of Veterinary Medicine, Alexandria University, Damanhour Branch, Egypt; 3 Department of Poultry Diseases and Hygiene, Faculty of Veterinary Medicine, Alexandria University, Edfina Branch, Egypt; 4 Graduate School of Pharmaceutical Sciences, Osaka University, Osaka, Japan; 5 Genome Information Research Center, Research Institute for Microbial Diseases, Osaka University, Osaka, Japan; 6 Health Scientific Hills, College of Life and Health Sciences, Chubu University, Aichi, Japan; 7 Faculty of Medicine, Thammasat University (Rangsit Campus), PathumThani, Thailand; Erasmus Medical Center, Netherlands

## Abstract

Highly pathogenic avian influenza A virus subtype H5N1 is currently widespread in Asia, Europe, and Africa, with 60% mortality in humans. In particular, since 2009 Egypt has unexpectedly had the highest number of human cases of H5N1 virus infection, with more than 50% of the cases worldwide, but the basis for this high incidence has not been elucidated. A change in receptor binding affinity of the viral hemagglutinin (HA) from α2,3- to α2,6-linked sialic acid (SA) is thought to be necessary for H5N1 virus to become pandemic. In this study, we conducted a phylogenetic analysis of H5N1 viruses isolated between 2006 and 2009 in Egypt. The phylogenetic results showed that recent human isolates clustered disproportionally into several new H5 sublineages suggesting that their HAs have changed their receptor specificity. Using reverse genetics, we found that these H5 sublineages have acquired an enhanced binding affinity for α2,6 SA in combination with residual affinity for α2,3 SA, and identified the amino acid mutations that produced this new receptor specificity. Recombinant H5N1 viruses with a single mutation at HA residue 192 or a double mutation at HA residues 129 and 151 had increased attachment to and infectivity in the human lower respiratory tract but not in the larynx. These findings correlated with enhanced virulence of the mutant viruses in mice. Interestingly, these H5 viruses, with increased affinity to α2,6 SA, emerged during viral diversification in bird populations and subsequently spread to humans. Our findings suggested that emergence of new H5 sublineages with α2,6 SA specificity caused a subsequent increase in human H5N1 influenza virus infections in Egypt, and provided data for understanding the virus's pandemic potential.

## Author Summary

Even though highly pathogenic avian H5N1 influenza viruses lack an efficient mechanism for human-human transmission, these viruses are endemic in birds in China, Indonesia, Viet Nam and Egypt. Hotspots for bird-human transmission are indicated in areas where human cases are more than 80% of total H5N1 influenza cases. Circulation among hosts may allow H5N1 virus to acquire amino acid changes enabling efficient bird-human transmission and eventually human-human transmission. The receptor specificity of viral hemagglutinin (HA) is considered a main factor affecting efficient transmissibility. Several amino acid substitutions in H5 virus HAs that increase their human-type receptor specificity have been described in virus isolates from patients, but their prevalence has been limited. In contrast, we show here that new H5 sublineages in Egypt have acquired a change in receptor specificity during their diversification in birds. We found that viruses in those sublineages exhibited increased attachment and infectivity in the human lower respiratory tract, but not in the larynx. Our findings may not allow a conclusion on the high pandemic potential of H5N1 virus in Egypt, but helps explain why Egypt has recently had the highest number of human H5 cases worldwide.

## Introduction

Since the emergence of highly pathogenic avian influenza virus subtype H5N1 (HPAI H5N1) in 1996, outbreaks have continued in a variety of domestic and wild birds as well as sporadic transmission to humans [1]. Over time, H5N1 viruses have diversified and are currently grouped into clades 0 to 9 according to the unified nomenclature system [2]. Since 2006, clade 2.2, which originated from a large outbreak in wild bird populations at Qinghai Lake in western China [3], [4], has spread rapidly over central Asia, Europe, the Middle East, and Africa [5], [6]. Clade 2.2 has further diversified forming the third-order clade 2.2.1 and three phylogenetically distinct sublineages (Ι, ΙΙ and ΙΙΙ) within clade 2.2 [7], [8].

Although the current H1N1 pandemic [9] may have diverted attention from the continuing worldwide circulation of H5N1 virus, the pandemic threat of H5N1 is still alarming. The cumulative number of confirmed human cases of H5N1 infection reported to the World Health Organization (WHO) to date is 504 with a 60% mortality [10]. According to the World Organization for Animal Health, HPAI H5N1 has become endemic in some areas where human cases constitute more than 80% of the total [10], indicating bird-human H5N1 virus transmission; e.g., China, Indonesia, Viet Nam and Egypt [11].

Since 2006, H5N1 viruses have spread across countries in western, eastern, and northern Africa, where viruses belonging to clade 2.2.1 and three sublineages (Ι, ΙΙ and ΙΙΙ) of clade 2.2 have been detected [7], [8]. As of October 2010, WHO has reported 114 laboratory-confirmed human cases on the African continent [10]. Egypt has experienced a relatively large number of human infections with 112 confirmed cases reported since 2006, when H5N1 was first identified in Egypt. In particular, the cumulative number since 2009 is notable: 61 confirmed cases in Egypt. The worldwide number since is also 112 cases. This indicates that the recent human H5N1 cases in Egypt are more than 50% of the total worldwide. The other 2 human cases of H5N1 virus infection in Africa were reported from Nigeria and Djibouti. The reason(s) for such a high number of human H5N1 cases in Egypt has not been elucidated.

Influenza viruses target glycosylated oligosaccharides that terminate in a sialic acid (SA) residue [12]–[14]. These residues are bound to glycans through an α2,3, α2,6, α2,8 or α2,9 linkage by sialyltransferases that are expressed in a tissue- and species-specific manner [15]–[17]. For example, human upper airway epithelia express mostly α2,6-linked SA (α2,6 SA) [18], whereas duck intestinal epithelia express mainly α2,3-linked SA (α2,3 SA) [19]. Efficient human-human transmission is necessary for influenza A virus to become pandemic. Although the determinants of efficient human-human transmission are not fully understood, it is believed that a change of receptor specificity from α2,3 SA, to which avian influenza A viruses preferentially bind, to α2,6 SA, to which human influenza viruses preferentially bind, is essential [12], [20], [21]. Although H5N1 viruses still lack the ability for efficient human-human transmission, the current prevalence of H5N1 might allow the virus to acquire mutations enabling α2,6 SA recognition. Thus, it is important to monitor the receptor binding affinity of H5N1 viruses in endemic areas and evaluate molecular mechanisms that might promote their pandemic potential.

In this study, we carried out a phylogenetic analysis of avian and human H5N1 viruses circulating in Egypt. The resulting virus phylogenetic tree indicated emergence of new H5 sublineages with each sublineage containing only or mostly human isolates, leading us to hypothesize that the HAs of these viruses might have acquired amino acid change(s) enabling α2,6 SA binding and resulting in the large number of human H5N1 cases in Egypt. Therefore, in this study we examined the receptor binding affinity of H5N1 viruses isolated in Egypt using sialylglycopolymers and human respiratory tract tissues, and assessed the effect of the amino acid changes in the HAs on viral replication in human airway epithelia *in vitro* and virulence in mice *in vivo*. We show here that these H5N1 viruses, during their spread in local bird populations, acquired mutations in their HAs that produced α2,6 SA binding affinity, providing a model for influenza virus phylogeny.

## Results

### Phylogeny of H5N1 viruses circulating in Egypt

We studied the evolution of H5N1 influenza viruses in Egypt by analyzing the sequences of 106 viruses isolated there from birds and humans between 2006 and 2009: 85 sequences were obtained from the National Center for Biotechnology Information (NCBI) database, and 21 sequences were newly obtained in this study. At the time of this investigation, these 106 sequences represented 40% of the complete and partial H5N1 virus sequences from Egypt in public databases. HPAI H5N1 emerged in Egypt first in poultry in 2006, swiftly spread to many species of birds in different geographic regions [11], [22], and was declared endemic in 2008 [11]. Human infections started shortly thereafter and reached 112 cases by October 2010 [1], [10].

**Figure 1:**
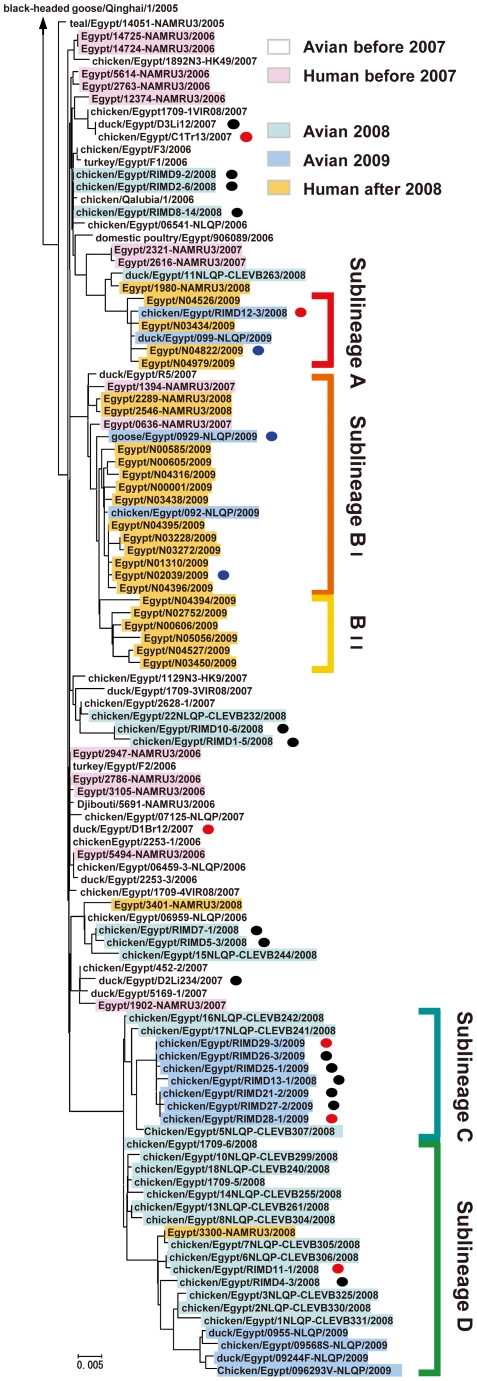
Phylogenetic tree of HA genes of H5N1 viruses isolated in Egypt. This tree includes published HA sequences of 85 H5N1 influenza A viruses isolated in Egypt, from the National Center for Biotechnology Information database (minimum sequence length 1,644 nt), and 21 HA sequences determined in this study (sequence length 1,707 nt). The newly analyzed sequences in this study are marked with a black circle. The strains whose HA sequences were determined in this study and were analyzed further for receptor binding specificity are marked with a red circle. The strains whose HA sequences were previously reported and were analyzed for receptor binding specificity in this study are marked with a blue circle. Colors are used to highlight virus strains with different hosts, isolation year and sublineage.

Phylogenetic analysis of the 106 H5N1 virus HA genes showed that all of these HA genes clustered in clade 2.2.1, with some of these viruses forming several new H5 sublineages (Figure 1). H5N1 isolates from 2006–2007 were interspersed throughout the phylogenetic tree, indicating rapid spread of the ancestral HA gene. In contrast, most human and avian isolates from 2008–2009 were clustered separately in distinct sublineages, denoted here as sublineages A, B (Ι, ΙΙ), C and D. These phylogenetic relationships indicated that during 2007–2008 the genetic diversity of H5 HA in Egypt increased dramatically and resulted in the establishment of distinct human and avian sublineages. Conversely, phylogenetic analysis of viral neuraminidase (NA) genes revealed that these genes were less divergent (Figure S1), with branches and tree topology different than the HA tree. The NA sequences formed a single monophyletic cluster which included the virus with the ancestral HA gene. These findings suggested that H5N1 viruses circulating in Egypt have diversified without significant genetic linkage, at least between the HA and NA genes.

### SA binding specificity of H5N1 viruses isolated in Egypt

The phylogenetic distribution of human and avian isolates in Egypt prompted us to investigate whether recent Egyptian isolates had an altered receptor binding specificity. To determine the α2,3 SA- and α2,6 SA-binding affinity of these isolates, we performed direct binding assays with SAα2,3Gal and SAα2,6Gal sialylglycopolymers [23], [24]. Six H5N1 isolates from outbreaks in Egypt during 2007–2009 were tested: A/duck/Egypt/D1Br12/2007 (EG/D1), A/chicken/Egypt/C1Tr13/2007 (EG/C1), A/chicken/Egypt/RIMD11-1/2008 (EG/11), A/chicken/Egypt/RIMD12-3/2008 (EG/12), A/chicken/Egypt/RIMD28-1/2009 (EG/28), and A/chicken/Egypt/RIMD29-3/2008 (EG/29). EG/D1 and EG/C1 were isolated from 2007 outbreaks, shared >99% homology with H5N1 viruses isolated in 2006, and in our phylogenetic tree did not form a sublineage or group with other H5N1 viruses isolated in Egypt, implying that they emerged before the establishment of new sublineages in Egypt and indicating that they were phylogentically close to the original H5N1 genotype in Egypt. The other four isolates belonged to the new H5 phylogenetic sublineages (Figure 1), indicating that they emerged during more recent H5N1 outbreaks.

Preliminary experiments to determine optimal binding assay conditions showed the importance of using appropriate virus titers (i.e., hemagglutination titers), because high virus titers produced exaggerated signals for the weakly binding glycopolymer (α2,6 SA) and low titers only detected binding to the high-affinity glycopolymer (α2,3 SA) (Figure S2). For example, EG/D1, which was expected to have a classical avian influenza virus α2,3 SA specificity, showed strong binding to α2,3 SA as expected, but also measurable binding to α2,6 SA when the virus titer was increased to 512 HAU. Conversely, EG/12, which was assumed to have increased α2,6 SA specificity because it clustered with human sublineage A strains, showed a complete loss of α2,6 SA binding with increasing dilution of the HA titer to 8 HAU. From these results, HA titers from 32 to 128 HAU appeared to be optimal for comparison of receptor binding specificity with our experimental conditions. Therefore, HA titers of all virus samples were adjusted to an HA titer of 64 HAU, relative to a reference EG/D1 sample, and used for the following binding assays.

**Figure 2:**
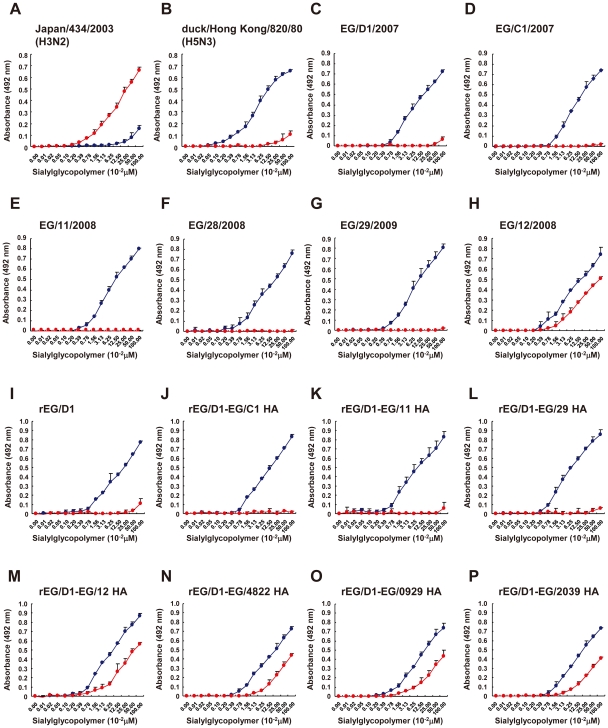
Receptor-binding specificity of H5N1 viruses isolated in Egypt. Direct binding of viruses to sialylglycopolymers containing either α2,3-linked (blue) or α2,6-linked (red) sialic acids was measured. (A) Seasonal human influenza H3N2 virus. (B) Avian influenza H5N3 virus. (C)–(H) Isolates from 2007–2009 outbreaks in Egypt. (I)–(P) Recombinant EG/D1 viruses with different HAs as indicated. Each data point is the mean ± SD of triplicate experiments.

EG/D1, EG/C1, EG/11, EG/28 and EG/29 viruses had binding specificity for α2,3 SA (Figure 2C–2G). The association constants are shown in Table S1. The binding patterns closely resembled the strong α2,3 SA binding specificity observed with an avian influenza H5N3 virus, A/Duck/Hong Kong/820/80 (Figure 2B). In contrast, EG/12 virus had appreciably increased binding to α2,6 SA, with binding to both α2,3 SA and α2,6 SA (Figure 2H). However, the EG/12 binding affinity for α2,6 SA was less than that of the seasonal human influenza virus A/Japan/434/2003 (Figure 2A). This was confirmed by direct binding assays using recombinant viruses generated by reverse-genetics: each recombinant virus contained one of the HA genes in a background of all of the other EG/D1 virus genes (denoted here as rEG/D1) (Figure 2I–2M). To investigate other sublineage A and B viruses, we synthesized the HAs of three H5N1 viruses isolated in Egypt: a bird isolate; A/goose/Egpt/0929-NLQP/2009 (EG/0929); and two human isolates; A/Egypt/N04822/2009 (EG/4822) and A/Egypt/N02039/2009 (EG/2039). The receptor specificities of these viruses were determined and showed that the H5 HAs of these recent isolates also had increased α2,6 SA binding (Figure 2N–2P). These results indicated differences in HA affinity to α2,6 SA among recent H5 isolates, together with an affinity to α2,3 SA.

### Identification of amino acid mutations in viral HAs enabling α2,6 SA binding

**Figure 3 ppat-1002068-g003:**
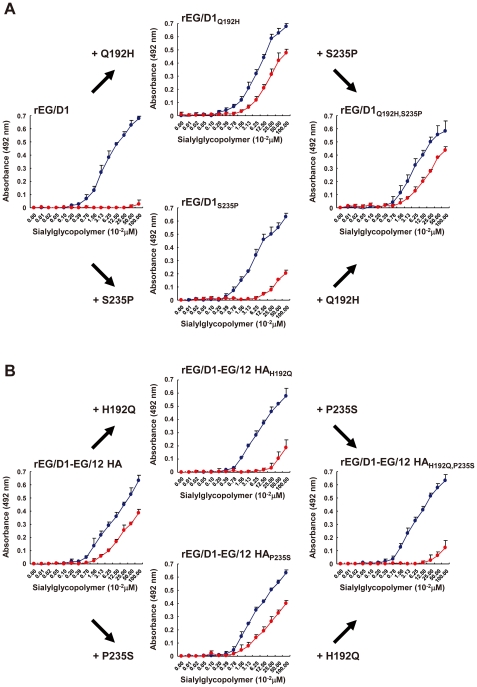
Effect of HA mutations in sublineage A viruses on receptor specificity of EG/D1 virus HA. (A) The two mutations found in the HAs of sublineage A viruses were introduced into the HA of EG/D1 virus as single and double mutations. (B) The reverse mutations were introduced into the HA of EG/12 virus. Direct binding to sialylglycopolymers containing either α2,3-linked (blue) or α2,6-linked (red) sialic acid was assayed. Mutations are indicated by subscripts. Each data point is the mean ± SD of triplicate experiments.

To identify mutations enabling α2,6 SA binding, we focused on viruses in sublineages A and B, to which most human isolates belonged. Comparison of 6 HA sequences of sublineage A viruses with 100 HA sequences of other H5 viruses isolated in Egypt identified two amino acid changes in the sublineage A virus HAs (Table 1): Q192H and S235P (H5 HA numbering). Introduction of the Q192H mutation into EG/D1 HA (denoted rEG/D1_Q192H_) markedly increased viral binding to α2,6 SA (Figure 3A). However, introduction of the S235P mutation into EG/D1 HA (denoted rEG/D1_S235P_) only slightly increased α2,6 SA binding. There was no synergistic effect with both mutations: the double mutant had similar α2,6 SA binding to that of the single Q192H mutant. In contrast, the H192Q mutation, but not the P235S mutation, in HAs of EG/12 (denoted rEG/D1-EG/12 HA_H192Q_) and EG/4822 decreased α2,6 SA binding (Figures 3B and S3A). These findings suggested that the Q192H mutation in H5N1 avian viruses increased the binding affinity of HA for the human receptor.

**Table 1:** Mutations in HA genes in H5 viruses in sublineages A, BI and BII.

Sublineage (no. of strains in sublineage)	Isolation year	Mutation in HA^a^	% of strains with mutation (no. of strains with mutation/total no. of strains)
A (6)	2008–2009	Q192H	100 (6/6)
		S235P	100 (6/6)
BI (19)	2007–2009	S120N	94 (18/19)
		129Δ	100 (19/19)
		I151T	100 (19/19)
BII (5)	2009	S120N	100 (5/5)
		129Δ	100 (5/5)
		I151T	100 (5/5)
		V210I	100 (5/5)

a H5 numbering, Δ denotes deletion.

**Figure 4 ppat-1002068-g004:**
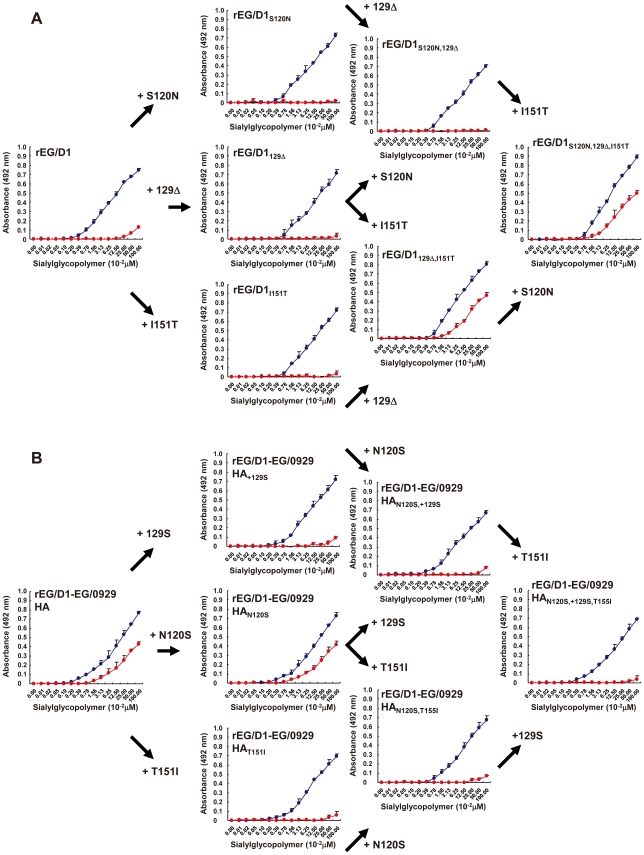
Effect of HA mutations in sublineage BI viruses on receptor specificity of EG/D1 HA. (A) The mutations found in sublineage BI viral HAs were introduced as single and multiple mutations into the HA of EG/D1 virus. (B) The reverse mutations were introduced into the HA of EG/0929 virus. Direct binding to sialylglycopolymers containing either α2,3-linked (blue) or α2,6-linked (red) sialic acid was measured. Mutations are indicated by subscripts. Each data point is the mean ± SD of triplicate experiments.

**Figure 5:**
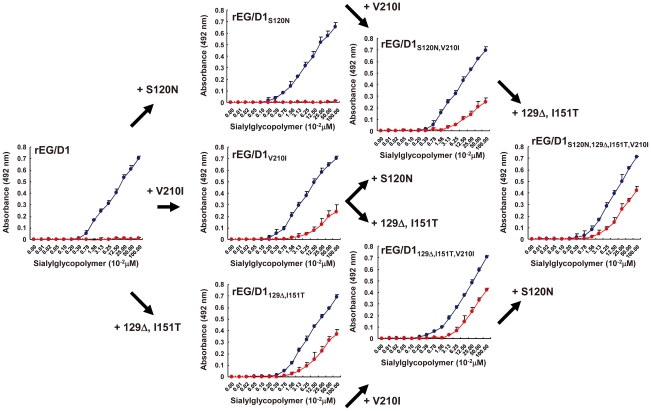
Effect of HA mutations in sublineage BII viruses on receptor specificity of EG/D1 HA. The mutations found in sublineage BΙΙ viral HAs were introduced as single and multiple mutations into the HA of EG/D1 virus. Direct binding to sialylglycopolymers containing either α2,3-linked (blue) or α2,6-linked (red) sialic acid was measured. Mutations are indicated by subscripts. Each data point is the mean ± SD of triplicate experiments.

The HA sequences of the 19 H5N1 viruses in sublineage BI (denoted sublineage A1 in a previous report [25]) differed from the 87 HA sequences of other H5N1 viruses isolated in Egypt at three HA amino acid residues: S120N, 129 deletion (Δ) and I151T (Table 1). When introduced as a single mutation into EG/D1 HA, none of these amino acid changes increased binding to α2,6 SA (Figure 4A). However, the 129Δ/I151T double mutation increased α2,6 SA binding. In contrast, both the 129S insertion and the T151I mutation in the HAs of EG/0929 and EG/2039 decreased α2,6 SA binding (Figures 4B and S3B). These results suggested that the 129Δ and I151T mutations acted synergistically to enable α2,6 SA binding by sublineage BI viruses. Sublineage BII viruses have four mutations: the three mutations found in sublineage BI viruses plus an additional V210I mutation (Table 1). When introduced as a single mutation in the HA of EG/D1, V210I partially increased α2,6 SA binding, but there was not an appreciable increase in binding in the V210I/129Δ/I151T triple mutant (Figure 5). These results suggested that the V210I mutation did not increase α2,6 SA binding in sublineage BII viruses above that of the 129Δ/I151T double mutation.

### Genetic properties of H5N1 viruses in sublineages A, BI and BII

The phylogenetic trees of sublineages A, BI and BII suggested human viruses in these sublineages emerged from avian viruses in these sublineages or closely related avian viruses (Figure 1). The amino acid changes in HA enabling α2,6 SA binding in sublineage A (Q192H) and sublineage BI viruses (129Δ/I151T) were not found in human H5N1 influenza viruses phylogenetically unrelated to sublineage A and B strains (data not shown), indicating that these mutations were associated with the phylogeny of avian H5N1 sublineage A and B viruses in Egypt. A database search of virus gene sequences posted since 2006 also revealed that the prevalence of these amino acid changes increased in human influenza virus HAs in Egypt concurrently with an increase in avian influenza viruses in Egypt (Table 2), although most of the recent avian influenza virus isolates were in sublineages C and D (Figure 1). In contrast, an increased prevalence has not been detected in either birds or humans in Asia. These findings suggested that H5N1 avian viruses in Egypt acquired binding affinity for α2,6 SA during viral diversification in local bird populations, which may have contributed to subsequent virus transmission to humans with higher efficiency.

**Table 2:** Prevalence of HA mutations characteristic of H5 sublineages A, BI and BII viruses in virus isolates from Egypt and Asia.

		% of strains showing mutation characteristic of sublineage isolated in (no. of virus strains analyzed)^a^^,^ b															
		Asia (495)								Egypt (260)							
		Birds (375)				Humans (120)				Birds (189)				Humans (71)			
		Isolation year (no. strains)				Isolation year (no. strains)				Isolation year (no. strains)				Isolation year (no. strains)			
Sublineage	Characteristic mutation in HA	2006(181)	2007(134)	2008(47)	2009(13)	2006(95)	2007(20)	2008(5)	2009(0)	2006(37)	2007(70)	2008(73)	2009(9)	2006(16)	2007(23)	2008(7)	2009(25)
A	Q192H	0	0	0	0	0	0	0	-	0	0	1.3	11.1	0	0	0	16.0
	S235P	93.9	98.5	97.8	100	100	100	100	-	0	1.4	5.4	11.1	0	4.3	42.8	24.0
BI	S120N	0.5	3.7	0	0	0	0	16.6	-	0	11.4	5.4	11.1	0	21.7	28.5	84.0
	129 Δ	0	0	0	0	0	0	0	-	0	8.5	5.4	22.2	0	21.7	28.5	84.0
	I151T	0	0.7	10.6	0	2.1	0	0	-	0	8.5	5.4	22.2	0	21.7	28.5	84.0
BII	V210I	1.6	2.2	21.2	0	0	0	0	-	0	0	0	0	0	0	0	20.0

a Percent of H5N1 viruses that have mutation characteristic of sublineages A, BI and BII for each geographic region, host and year.

b Sequence information from the National Center for Biotechnology Information database in addition to sequences analyzed for this study.

- denotes no sequence information available.

### Attachment of rEG/D1 viruses in the human respiratory tract

**Figure 6:**
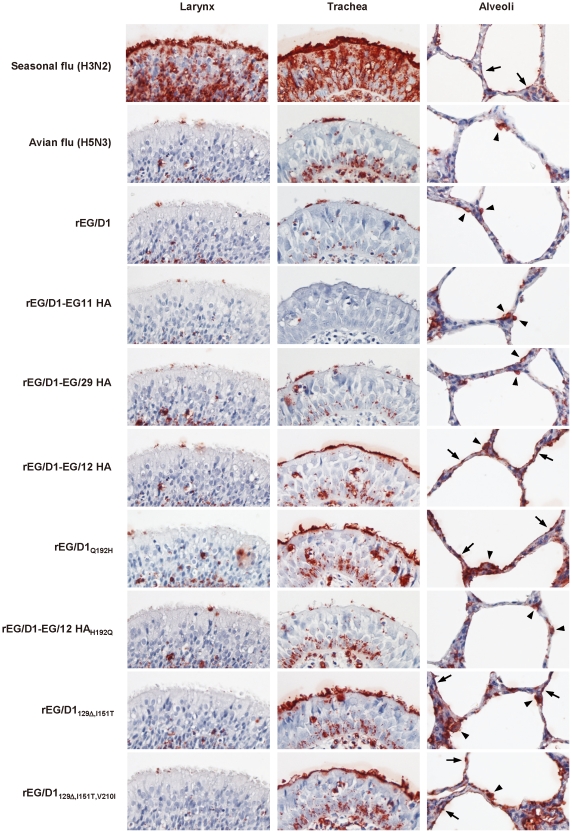
Attachment of rEG/D1 viruses to tissues of the human respiratory tract. The attachment patterns of A/Japan/434/2003 (H3N2), A/Duck/Hong Kong/820/80 (H5N3), and eight rEG/D1 viruses (rEG/D1, rEG/D1-EG/11 HA, rEG/D1-EG/29 HA, rEG/D1-EG/12 HA, rEG/D1_Q192H_, rEG/D1_129Δ,I151T_, rEG/D1_129Δ,I151T,V210I_ and rEG/D1-EG/12 HA_H192Q_) to fixed human larynx, trachea and alveoli tissue sections were examined by histochemistry. Attached viruses were stained red. Arrows and arrow-heads indicate type I and type ΙΙ pneumocytes, respectively. The panels were chosen to reflect the attachment pattern in each tissue section as much as possible.

To investigate whether mutations in avian virus HAs enabling α2,6 SA binding function with similar specificity in the human respiratory tract, the attachment pattern of selected viruses to fixed tissues of the human upper and lower respiratory tract (i.e., larynx, trachea and alveoli) was determined by histochemistry. Histochemical analysis can provide clinically relevant data on virus attachment in human airway epithelia [26], [27] and on the glycan topologies that influenza viruses target for cell-specific infections in airway epithelia [28], [29]. Human H3N2 virus, which was used as a control, attached extensively to ciliated epithelial cells in the larynx and trachea and, to lesser degree, to alveolar cells (type Ι pneumocytes; Figure 6). In contrast, rEG/D1, rEG/D1-EG/11 HA and rEG/D1-EG/29 HA attached predominantly to alveolar cells (type ΙΙ pneumocytes), with little attachment in larynx and trachea, as found for avian H5N3 virus. The attachment pattern of rEG/D1-EG/12 HA was different from the classical avian pattern found for H5N3: little attachment to larynx, moderate attachment to trachea, and significant attachment to alveoli (both type Ι and ΙΙ pneumocytes). The attachment patterns of the rEG/D1_Q192H_, rEG/D1_129Δ,I151T_ and rEGD1_129Δ,I151T,V210I_ mutants were similar to that of rEG/D1-EG/12 HA. However, all three mutant viruses attached less abundantly to trachea than the H3N2 virus. Also, rEG/D1-EG/12 HA_H192Q_ showed an attachment pattern similar to that of rEG/D1, with rare attachment to trachea. We also performed virus histochemistry on sialidase-treated sections, which abrogated all staining confirming that the viruses in this study did not bind to non-sialic acid residues (Figure S4). Although not quantitative, these results indicated that mutations enabling α2,6 SA binding are clinically significant in affecting the affinity of HA for receptors in the human respiratory tract.

### Replication of rEG/D1 viruses in a human airway epithelial culture

**Figure 7:**
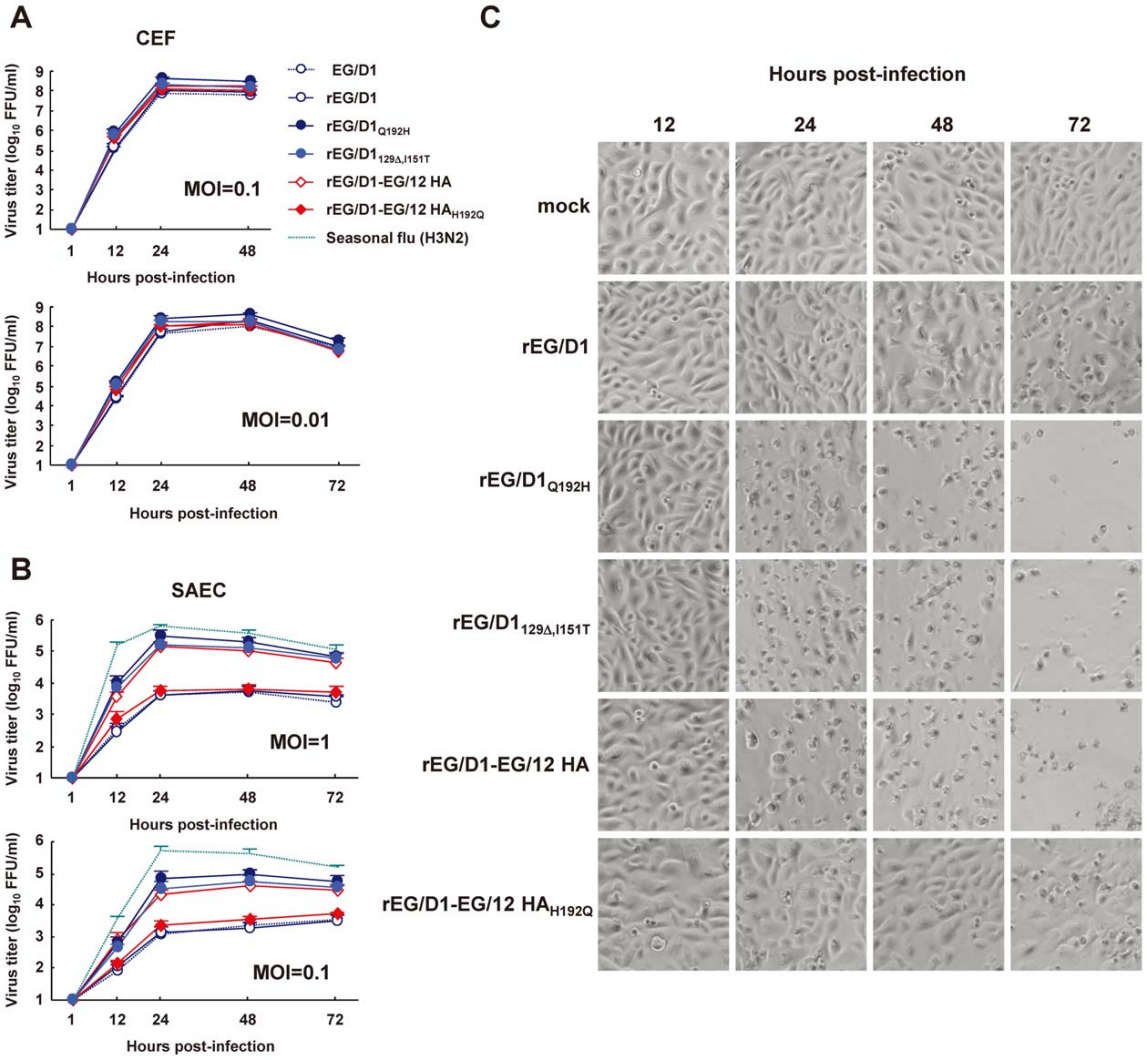
Growth kinetics of rEG/D1 viruses in avian cells and human cells. (A) CEF cells were infected in triplicate with parental EG/D1 and five rEG/D1 viruses (rEG/D1, rEG/D1_Q192H_, rEG/D1_129Δ,I151T_, rEG/D1-EG/12 HA and rEG/D1-EG/12 HA_H192Q_) at an MOI of 0.1 or 0.01. (B) Human SAEC cells were infected in triplicate with the viruses at an MOI of 1 or 0.1. The culture supernatants were harvested at the indicated times and assayed for focus-forming units on CEF cells to determine the progeny virus titer (log_10_ FFU/ml). Each data point in (A) and (B) is the mean ± SD of triplicate experiments. (C) Phase contrast microscopy of morphological changes in SAEC cells infected by the indicated viruses at an MOI of 0.1 and examined at the indicated times post-infection.

To examine whether the HA mutations enabling α2,6 SA binding also affected virus replication in human airway cells, we studied virus growth in primary human small airway epithelial cells (SAEC) by infecting these cells with selected recombinant viruses and human H3N2 virus, which was used as a control, at a multiplicity of infection (MOI) of 1 or 0.1 and monitoring viral growth kinetics and cytopathicity for 72 h post-infection. For comparison, we studied viral growth kinetics in chicken embryo fibroblast (CEF) cells infected at an MOI of 0.1 or 0.01. All viruses replicated well in CEF cells and produced >10^7^ focus-forming units (FFU)/ml at 24 and 48 h post-infection. The difference in titers of these viruses was <1 log FFU/ml at each time point, indicating that all of the viruses replicated equally well in avian-derived cells (Figure 7A). These results confirmed that there was no incompatibility between EG/12 HA and EG/D1 NA or between mutated EG/D1 HA and EG/D1 NA in the recombinant viruses generated for this study (compare the kinetics of parental EG/D1 and rEG/D1 viruses in Figure 7A). In contrast, in SAEC cells (Figure 7B), rEG/D1_Q192H_, rEGD1_129Δ,I151T_ and rEG/D1-EG/12 HA replicated more efficiently than rEG/D1 and rEG/D1-EG/12 HA_H192Q_, with slight differences in their growth, and a final virus titer of rEG/D1_Q192H_ > rEG/D1_129Δ,I151T_ > rEG/D1-EG/12 HA. These viruses replicated in SAEC cells and reached titers more similar to those of human H3N2 virus than of parental EG/D1, especially at a higher inoculum. The difference in virus growth kinetics correlated with cytopathicity in SAEC cells: rEG/D1_Q192H_, rEG/D1_129Δ,I151T_ and rEG/D1-EG/12 HA produced more severe cytopathic effects and resulted in more detachment of infected cells at 24, 48 and 72 h post-infection than rEG/D1 and rEG/D1-EG/12 HA_H192Q_ (Figure 7C). These results indicated that the Q192H mutant and the 129Δ/I151T double mutant produced a substantial viral growth advantage in human airway epithelial cells.

### Effect of HA mutations on virulence of rEG/D1 viruses in mice *in vivo*

**Figure 8:**
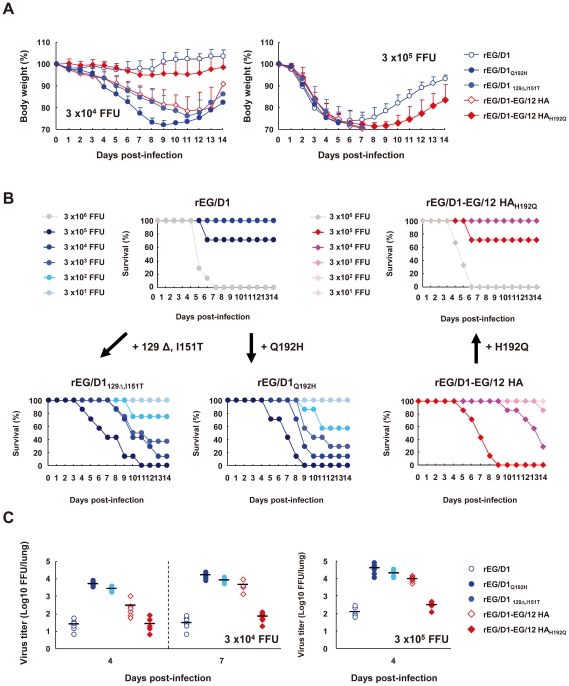
Mortality and weight loss of mice infected with rEG/D1 viruses. Six-week-old BALB/c mice (7–8 mice per group) were inoculated intranasally with the indicated doses of rEG/D1, rEG/D1_Q192H_, rEG/D1_129Δ,I151T_, rEG/D1-EG/12 HA and rEG/D1-EG/12 HA_H192Q_. (A) Body weight of infected mice was monitored up to 14 d post-infection. Mean percent body weight change (±SD) for each group of mice is shown. (B) Survival of mice inoculated with rEG/D1 viruses. Mortality was calculated including mice that were sacrificed because they had lost more than 30% of their body weight. (C) Virus titers in lungs of mice infected with 3×10^4^ or 3×10^5^ FFU rEG/D1 at the indicated times post-infection. Circles and diamonds indicate values in individual mice.

To assess the effect of enhanced α2,6 SA binding on pathogenicity of H5N1 isolates from Egypt, BALB/c mice were inoculated intranasally with different dilutions of selected recombinant viruses. Mice inoculated with 3×10^4^ FFU rEG/D1_Q192H_, rEG/D1_129Δ,I151T_ or rEG/D1-EG/12 HA showed considerable weight loss (Figure 8A). In contrast, mice inoculated with 3×10^4^ FFU rEG/D1 or rEG/D1-EG/12 HA_H192Q_ showed no clinical effects during the 14 d observation period, and most mice infected with 3×10^5^ FFU of these viruses survived. The lethality of rEG/D1_Q192H_, rEG/D1_129Δ,I151T_ and rEG/D1-EG/12 HA was substantially higher: the MLD_50_ was 8.8×10^2^ FFU for rEG/D1_Q192H_, 1.5×10^3^ FFU for rEG/D1_129Δ,I151T_ and 1.3×10^4^ FFU for rEG/D1-EG/12 HA (Figure 8B), >50 times less than the MLD_50_ of 5.9×10^5^ FFU for both rEG/D1 and rEG/D1-EG/12 HA_H192Q_. Consistent with this result, the virus yield in lungs of mice infected with 3×10^4^ FFU of the three viruses was >10-fold higher 4 d post-infection and >110-fold higher 7 d post-infection, and at a dose of 3×10^5^ FFU was >70-fold higher 4 d post-infection than with parental rEG/D1 virus (Figure 8C).

**Figure 9:**
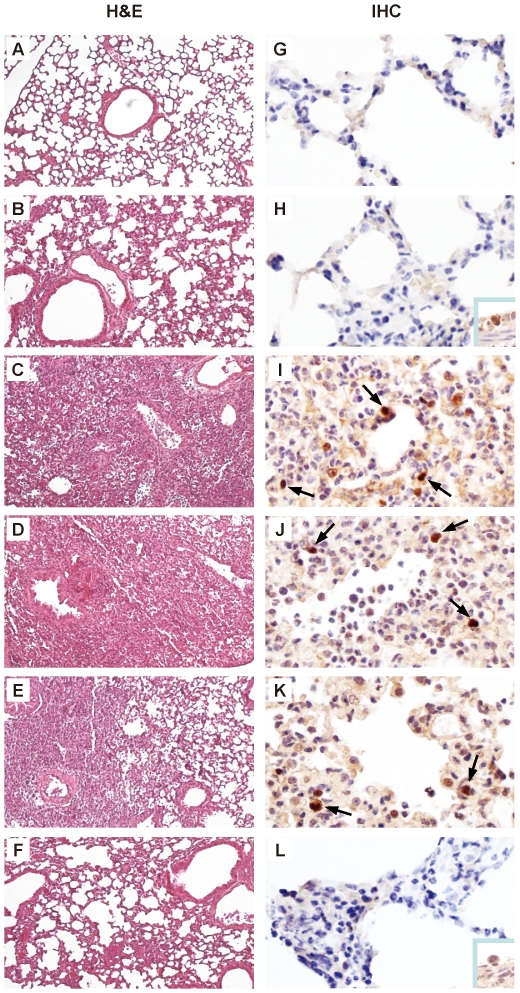
Histopathology and immunohistochemistry in lung tissues of mice infected with rEG/D1 viruses. Photomicrographs of hematoxylin-and-eosin (H&E) stained and immunohistochemically (IHC) stained lung sections from mice infected with 3×10^4^ FFU rEG/D1 viruses 7 d post-infection are shown as follows. (A) and (G) mock-infected. (B) and (H) rEG/D1-infected. (C) and (I) rEG/D1_Q192H_-infected. (D) and (J) rEG/D1_129Δ,I151T_-infected. (E) and (K) rEG/D1-EG/12 HA-infected. (F) and (L) rEG/D1-EG/12 HA_H192Q_-infected. In the IHC-stained tissues, viral antigen is stained deep brown on a hematoxylin-stained background (arrows). In mice infected with rEG/D1 and rEG/D1-EG/12 HA_H192Q_, positive staining was detected sporadically in the bronchiolar epithelium (insert).

Lungs of mice infected with 3×10^4^ FFU viruses were examined by histopathology at 7 d post-infection. Mice infected with rEG/D1_Q192H_, rEG/D1_129Δ,I151T_ or rEG/D1-EG/12 HA had much more dramatic pathological changes in their pulmonary airways and parenchymal tissues. The lungs had moderate to severe bronchiolar necrosis and alveolitis with associated hyperplasia, pulmonary edema and inflammatory cell infiltrates (Figure 9C–9E). In contrast, lung pathology of rEG/D1 and rEG/D1-EG/12 HA_H192Q_ infected mice showed signs of limited lymphohistiocytic cell extravasations (Figure 9B and 9F). Mock-infected mice did not have lesions in their lungs (Figure 9A). H5 antigen was more extensively detected by immunohistochemistry in the alveolar area of lungs infected with rEG/D1_Q192H_, rEG/D1_129Δ,I151T_ or rEG/D1-EG/12 HA than in lungs infected with rEG/D1 and rEG/D1-EG/12 HA_H192Q_ (Figure 9G–9L). Weak antigen staining was only rarely detected in the bronchiolar area in lungs of mice infected with rEG/D1 and rEG/D1-EG/12 HA_H192Q_ (see insert in Figure 9H and 9L). Therefore, the difference in lethality in mice infected with these viruses was grossly correlated with the growth kinetics and cytopathicity of the viruses in human airway epithelial cells. Collectively, these results indicated that enhanced receptor specificity *in vivo* enabled rEG/D1_Q192H_, rEG/D1_129Δ,I151T_ and rEG/D1-EG/12 HA to infect mice at lower titers than rEG/D1 and rEG/D1-EG/12 HA_H192Q_.

### Effect of mutations on structural changes in EG/D1 HA

**Figure 10:**
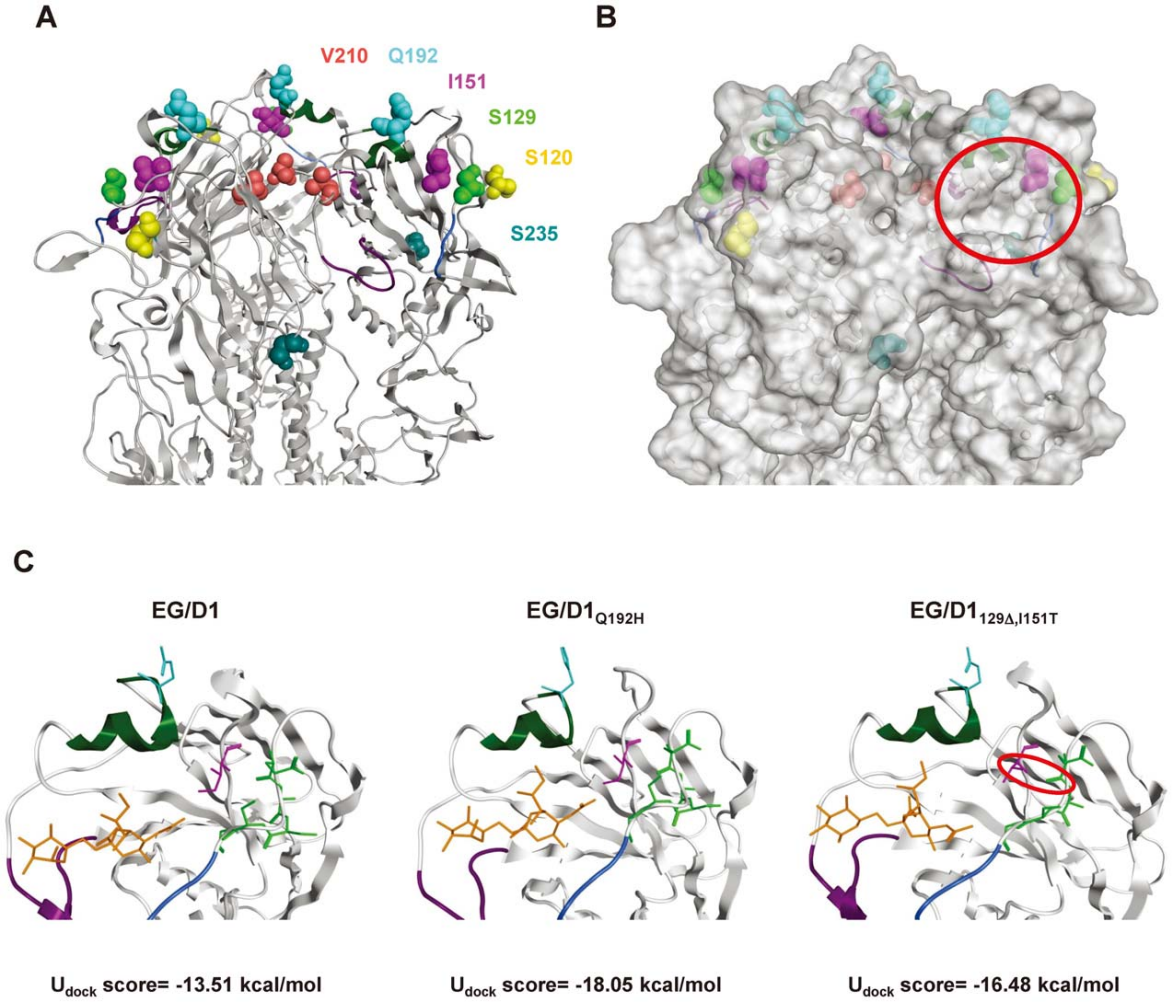
Analysis of receptor docking modes of EG/D1 HA and HA mutants. Structural models of H5 HA. (A) Ribbon model of EG/D1 HA. The trimeric globular-head region is shown. Key residues in our analysis are shown in a colored space-filling model. Receptor binding domains are colored blue (130 loop), green (190 helix) and purple (220 loop). (B) Molecular surface of EG/D1 HA. The red circle indicates the receptor binding pocket. (C) Docking models for EG/D1, EG/D1_Q192H_ and EG/D1_129Δ,I151T_ HA with a human-type receptor analog (PDBID code 1MQN). Residues 127E, 128A, 130S and 131G are colored green, as is 129S, and the other residues and domains are displayed in the same colors as above. An additional hydrogen bond between E127 and T151 is indicated in the red circle. The U_dock_ scores of the corresponding complexes are shown at the bottom.

To investigate the structural basis for the changes in human receptor-binding specificity in viruses in the new sublineages, we generated models of the HA structures of EG/D1, EG/D1_Q192H_ and EG/D1_129Δ,I151T_ from the crystal structure of the HA of A/Vietnam/1194/04 (H5N1) (Protein Data Bank ID (PDBID) code 2IBX) [24], and performed a docking study with these models and two types of ligands, SAα2,3Gal (PDIBID code 1MQM) and SAα2,6Gal (PDIBID code 1MQN). In our modeling, HA residues 120, 210 and 235 were distant from the receptor binding sites in the EG/D1 HA structure, whereas residues 129, 151 and 192 were located around them (Figure 10A and 10B). A Gln192 to histidine mutation (and a Gln192 to arginine mutation) generated a positively-charged side chain in the HA carbon backbone at this position, which has been reported [24] to stabilize contact of SAα2,6 Gal-terminated polysaccharides with H5 HA by forming a hydrogen bond with human receptor moieties (also see Discussion below). In addition, deletion of Ser129 led to a hydrogen bond between side chains of the HA carbon backbone at Glu127 and Thr151, affecting orientation of the 130-Loop (Figure 10C). Therefore, the double 129Δ/I151T mutation might affect the contact angle between human-type receptor ligands and viral HA.

In our simulation, the U_dock_ scores of the complexes between the SAα2,6 human-type receptor ligand and EG/D1, EG/D1_Q192H_ and EG/D1_129Δ,I151T_ HA were −13.51, −18.05 and −16.48 kcal/mol, respectively (Figure 10C). Therefore, the U_dock_ scores of the complexes bound to EG/D1_Q192H_ and EG/D1_129Δ,I151T_ HA were more negative than with parental EG/D1 HA, indicating more energetically stable interactions of the mutant HAs with the human receptor analog. In contrast, the U_dock_ scores of the complexes between the SAα2,3 avian-type receptor ligand and EG/D1, EG/D1_Q192H_ and EG/D1_129Δ,I151T_ HA were relatively similar (−14.19, −15.27 and −12.16 kcal/mol, respectively), with the U_dock_ scores of the complexes bound to EG/D1_Q192H_ and EG/D1_129Δ,I151T_ HA not appreciably more negative than with EG/D1 HA. These results indicated that HAs of the viruses in the new sublineages have structurally and energetically more stable conformations for binding human receptors.

## Discussion

In this study of H5N1 avian and human influenza viruses isolated in Egypt, we found that these viruses clustered in several new H5 sublineages, with a higher than expected binding affinity for α2,6 SA, and identified the amino acid mutations responsible for this expanded receptor specificity. Our phylogenetic analyses also indicated that these viruses emerged during 2007–2008 outbreaks in Egypt. This time overlaps with or slightly precedes an increase in the number of human cases of H5N1 virus infection in Egypt [1], [10].

HA plays an important role in the attachment of influenza viruses to host cells and, therefore, influences viral host range and pathogenicity [12], [30]–[32]. In this study of H5N1 virus (clade 2.2.1), we found that an HA Q192H single mutation or a 129Δ/I151T double mutation increased viral binding to α2,6 SA and increased infection in human airway epithelia. Previous assays [24] of A/Vietnam/3028ΙΙ/04 virus (clade 1) and A/chicken/Indonesia/N1/05 (clade 2.1) binding to sialylglycopolymers found that an HA Q192R mutation enhanced binding to α2,6 SA. The Q192H mutation identified in this study was at the same residue as the Q192R mutation in the A/Vietnam/3028ΙΙ/04 and A/chicken/Indonesia/N1/05 viruses, suggesting that these two mutations produced a similar conformational change in HA. These structural changes agreed fairly well with simulation data that the mutation at this position in an H5 HA model electrostatically enhanced HA binding affinity to human-like glycan [33]. The Q192H mutation was not present in H5 HAs in 375 avian influenza viruses and 120 human influenza viruses isolated in Asia, including clade 1, 2.1, 2.2 and 2.3 viruses (Table 2). We examined codon usage in HA of 495 H5 isolates from Asia and 254 isolates from Egypt with Q at residue 192 and found that all of these viruses encode 192Q using codon CAA. This result indicates that an amino acid change from Q to H or R at residue 192 required a one nucleotide change (CAA to CAT/CAC (H) or CGA(R)). The higher frequency of a transversion to encode H may have enabled such a mutation to occur more frequently in HAs of H5 viruses in Egypt.

Since the HA Q192R substitution might be selected during viral growth in a human patient and enhance α2,6 SA binding in the human respiratory tract [24], we constructed rEG/D1 with this HA substitution and found its α2,6 SA binding affinity similar to or slightly greater than rEG/D1_Q192H_ and rEG/D1_129Δ,I151T_ (data not shown). In addition, deletion of HA residue 129 was not found in any of the H5 HAs of the 495 Asian isolates examined, and the I151T substitution was only detected in H5 HAs isolated from 6 birds and 2 human patients in Asia (1.6% and 1.7% prevalence, respectively). H5 HA residues 129 and 151 make atomic contact with sialoglycosides [34]. We showed here that the 129Δ mutation generates a new hydrogen bond between Glu127 and Thr151, resulting in conformational changes around the binding pocket (Figure 10C). This effect around the glycosidic bond in H5 HAs seems to be unique to the viruses isolated in Egypt. We also searched for similar mutations (Q192H and the double 129Δ/I151T mutation) in 4507 avian influenza viruses with HAs H1–13 and H16 and found that only 3 bird isolates had these mutations (Table S2): Quail/Nanchang/12-340/2000 (H1N1), Turkey/Minnesota/40550/1987 (H5N2), and Ruddy turnstone/Delaware/2762/1987 (H11N2). Such mutations were not present in any of the H1, H2 or H3 HAs of the human isolates in the early years of the Spanish flu (1918), Asian flu (1957), Hong Kong flu (1968) and Russian flu (1977) pandemics, in which these avian subtypes crossed the species barrier to humans. However, it is noteworthy that most of the viruses in this study that clustered in sublineage B were reported to have evolved towards an H1N1-like receptor usage, to efficiently replicate in the upper respiratory tract, and that structural properties of the receptor binding sites of Spanish flu viruses and sublineage B viruses are much closer to each other than to other H1N1 and H5N1 viruses [35]. In contrast, a conformational change in HA due to S235P and S120N mutations was not observed in our structural model: these were also shown not to increase HA affinity for α2,6 SA by direct binding assays (data not shown).

Our data suggested that H5N1 viruses from Egypt had acquired amino acid mutations enabling α2,6 SA binding during their transmission among birds, not during viral growth in human patients. First, avian isolates were at the base and within branches of the phylogenetic tree of new sublineages A and B, and clustered closely with human isolates (Figure 1). Moreover, all of the avian isolates already had identical mutations that contributed to binding affinity for the human-type receptor (Tables S3, S4, S5). Second, critical amino acid mutations involved in α2,6 SA recognition (Q192H and the double 129Δ/I151T mutation) were not found in any of the H5 HAs from human isolates phylogenetically unrelated to sublineages A and B. Therefore, it is unlikely that viruses with these mutations were newly selected during viral growth in humans. Third, all viruses examined here exhibited a classical avian α2,3 SA binding affinity and replicated efficiently in CEF cells, suggesting that these viruses had retained HAs for efficient transmission among birds (Figure 7A).

Several amino acid mutations that increase α2,6 SA binding affinity of H5 virus HAs have recently been described in human isolates [23], [24], [36]. It is possible that such mutations were selected in humans and played an important role in viral recognition of human-type receptors. However, there have been only limited reports of those mutations in H5 HAs in infections in human patients [23], [24], [36]. Considering that the mutations in H5 HAs in birds identified here have been found in some population of birds in the vicinity of humans, such viral mutations emerging in birds may be as important risk factors for human H5N1 infections as those mutations emerging in viruses infecting humans. Thus far, there have been few reports of HPAIV in bird populations with increased affinity for α2,6 SA [37].

At present, the determinants of efficient human-human transmission by avian influenza viruses are not completely understood [21], [38]. It is generally thought that both a change in receptor specificity from α2,3 SA to α2,6 SA and the resultant shift in infection to the upper respiratory tract are essential [39]. However, most amino acid mutations in H5 HAs that have been reported to increase α2,6 SA binding have not conferred a complete change in receptor specificity in the original virus genetic background [23], [24], [40], [41]. But, Chutinimitkul *et al.* have recently reported that some mutations can cause a complete change in the A/Indonesia/5/05 background [42]. Increased α2,6 SA binding affinity and reduced α2,3 SA binding affinity was also observed among North American lineage H7 viruses isolated in 2002–2004 [37]. In contrast, we found that all of the H5N1 viruses in this study retained the classical avian α2,3 SA binding affinity (Figures 2–5). Histochemistry using human tissues also found that viruses in this study with avian H5 HA mutations had little attachment to the larynx, but moderate attachment to trachea and abundant attachment in alveoli (Figure 6), whereas human H3N2 virus extensively bound to both the larynx and trachea. These results suggested that H3 and H5 viruses recognized more complex glycan topologies, which have not yet been fully elucidated in human airway epithelia [28], [29]. This conclusion is in agreement with the suggestion that H5N1 viruses attach to receptors in the human upper respiratory tract that are not detected by lectin histochemistry and with data that H5N1 viruses can productively replicate in ex vivo cultures of human nasopharyngeal tissues [43].

These findings suggest that currently circulating H5N1 viruses in Egypt lack gene products for efficient human-human transmission, even though they have caused a relatively large number of human cases in Egypt. Indeed, most human infections resulted from direct exposure to H5N1 virus-infected poultry or poultry products and no sustained human-human transmission has been documented to date in Egypt [1], [44]. It should be noted that our findings do not allow determination of the potential for an H5N1-derived pandemic virus in Egypt. However, the emergence of sublineage A and B H5N1 viruses is a possible contributing factor to Egypt recently having the highest number of human H5N1 influenza virus cases in the world, with repeated avian infections increasing the probability of avian-human transmission. To our knowledge, this is the first report identifying amino acid changes in H5 HA responsible for an increase in human H5N1 infections in an endemic area.

Mice have been an animal model for studying influenza [45]–[47]. In this study, we found that the HA mutations enabling α2,6 SA binding enhanced viral virulence in BALB/c mice (Figure 8). These results are consistent with a previous report on different influenza viruses and different HA amino acid residues in a ferret model [48]. Reports on lectin histochemistry showed that BALB/c mice express both α2,3 and α2,6 SA in airway epithelia, with α2,3 SA specifically expressed in the upper respiratory tract and α2,6 SA expressed in pulmonary parenchyma [49]. Previous histochemistry report on seasonal human influenza viruses H3N2 and H1N1 showed rare attachment to mouse type I pneumocytes, indicating the presence of glycan topologies in alveoli to which influenza viruses with α2,6 SA binding affinity attach [27]. In this study, recombinant avian H5 viruses with single and double point mutations that should affect receptor binding were found to have acquired α2,6 SA binding affinity, and the resultant expansion of receptor specificity *in vivo* contributed to enhanced virulence in mice. Indeed, virus titers in the lungs of mice infected with the mutant viruses were more than one log higher than in mice infected with the parental virus (Figure 8C), corresponding to severe histopathological changes (Figure 9). These results were consistent with histochemistry showing that the mutants acquired enhanced attachment affinity to human type I pneumocytes (Figure 6). Type I pneumocytes comprise 96% of the alveolar surface area, which is extremely thin, thereby minimizing the diffusion distance between the alveolar air space and pulmonary capillary blood [50]. Therefore, viral binding specificity for this cell type has implications for the development of pneumonia. However, other factors also need to be considered, such as the low similarity of the SA expression pattern in mice relative to that in humans [26], [27]. Thus, it would be of interest to determine the effect of the substitutions in HA described here on virus virulence in the ferret model, which is a more suitable animal model for human H5N1 viral pneumonia [26], [27]. Our studies also found that EG/D1, an ancestral strain of currently circulating H5N1 viruses in Egypt, was not highly pathogenic in mice, as indicated by an MLD_50_ >10^5^ FFU (Figure 8B). Avian and human H5N1 viruses in Egypt, including EG/D1, encode PB2-627Lys, which reportedly enhances the host range and virulence of influenza viruses [30], [47], [51]. The results of this study indicate that this amino acid residue alone does not provide sufficient replicative advantage in mammals for the influenza viruses (clade 2.2.1) in Egypt, although it may be a prerequisite for H5N1 virus virulence in mammalian hosts.

The mechanism underlying the emergence of H5N1 viruses in Egypt with both α2,3 SA and α2,6 SA binding affinities is unclear. Some H7 viruses isolated in North America from 2002–2004 showed a marked decrease in α2,3 SA binding together with increased binding to glycans with α2,6 SA [37], and several H5N1 field isolates (clade 2.3.4) in the Lao People's Democratic Republic in 2007–2008 had reduced binding to α2,3 SA receptors [52]. In contrast, H5 viruses isolated in Egypt have retained the classical avian α2,3 SA binding affinity (Figures 2–5). Previous studies have shown passage of H5N1 viruses through land-based poultry as a possible mechanism for emergence of dual receptor specificity [17], [53]. However, most bird isolates in Egypt, found to be clustered in sublineages A and B in this study, were recently reported to be derived from domestic waterfowl, not from land-based poultry [54]. In addition, these H5N1 viruses showed an appreciably different attachment pattern in the human respiratory tract than that of typical avian viruses (Figure 6). Therefore, the binding properties of H5 viruses in Egypt may be the result of geographic and cultural factors that have yet to be identified.

Egypt has a relatively large number of human cases of H5N1 virus infection, and the highest number of cases worldwide since 2009 [1], [10]. The influenza virus phylogenetic tree suggests that sublineages A and B, the focus of this study, emerged during virus diversification in birds. At present, viruses grouped in sublineages C and D are widely disseminated across Egypt. Therefore, it remains possible that repeated circulation in birds would allow sublineage C and D viruses to acquire amino acid change(s) other than those identified here that could enable increased α2,6 SA binding affinity, although the amino acid mutations identified here may be useful markers in assessing H5N1 field isolates for their potential to infect humans. Since clade 2.2 appeared in Egypt in 2006, Egypt has had a single known introduction of a clade 2.2.1 H5N1 virus. Neither introduction of other phylogenetically distinct sublineages of clade 2.2 (I, II and III) nor reassortment events between the sublineages, as detected in neighboring Nigeria [55]–[57], have been documented in Egypt [7], [58]. Such events also were not observed in our phylogenetic analyses of HA and NA genes (Figures 1 and S1). However, introduction of these sublineages into Egypt could accelerate the evolutionary dynamics of H5N1 virus. Moreover, all Egyptian viruses (clade 2.2.1), which emerged during the 2005 Qinghai Lake outbreak in China [3], [4], have mammalian-type PB2-627Lys [30], [47], [51], implying the potential for evolution to a pandemic virus. Therefore, there is a critical need for continued surveillance of birds to monitor receptor specificities of H5N1 field isolates in Egypt as well as the pandemic potential of these strains.

## Materials and Methods

### Ethics statement

All animal studies were conducted under the applicable laws and guidelines for the care and use of laboratory animals in the Research Institute for Microbial Diseases, Osaka University, approved by the Animal Experiment Committee of the Research Institute for Microbial Disease, Osaka University, as specified in the Fundamental Guidelines for Proper Conduct of Animal Experiment and Related Activities in Academic Research Institutions under the jurisdiction of the Ministry of Education, Culture, Sports, Science and Technology, Japan, 2006.

### Virus isolation and preparation

During outbreaks of highly pathogenic avian influenza in Egypt from January 2007 to February 2009, 27 nasopharyngeal swab and tissue samples (lung and trachea) were collected from sick or dead chickens and ducks from commercial farms and backyard farms. Of these samples, 21 were identified as H5-positive by reverse transcription-polymerase chain reaction (RT-PCR) and selected for virus isolation. Twenty viruses were eventually isolated by single passage in the allantoic cavity of 11-day-old embryonated chicken eggs. The allantoic fluids were then harvested and stored as seed viruses at −80°C. Laboratory strains A/Duck/Hong Kong/820/80 (H5N3) and human influenza A virus A/Japan/434/2003 (H3N2) were kindly provided by Yoshinobu Okuno, Kanonji Institute, The Research Foundation for Microbial Diseases of Osaka University, Kagawa, Japan. For subsequent studies, allantoic fluids were pre-cleared by centrifugation at 3,000 rpm for 20 min and filtration through 0.45 µm filters, and viruses were then purified by centrifugation at 25,000 rpm for 2 h through 20% and 60% sucrose. After collection of the virus-containing fractions, viruses were suspended in PBS and pelleted by centrifugation at 25,000 rpm for 2 h. Virus pellets were resuspended in PBS and aliquots were stored as working stocks at −80°C. Virus titers were assayed as FFU by focus-forming assays [59] on CEF cells for avian influenza viruses and on MDCK cells for human H3N2 virus. All experiments with live H5N1 viruses were performed in Biosafety Level 3+ (BSL 3+) conditions at Osaka University, as approved for work with these viruses by the Ministry of Agriculture, Forestry and Fisheries, Japan.

### Cells

CEF cells were prepared from 11-day-old embryonated eggs. MDCK cells were purchased from the Riken BioResource Center Cell Bank (http://www.brc.riken.jp/lab/cell/english/). These cell lines were maintained in Dulbecco's Modified Eagle's Medium supplemented with 10% heat-inactivated fetal calf serum at 37°C in a humidified atmosphere of 95% air and 5% CO_2_ as described previously [60]. Human primary SAEC cells were purchased from the Lonza Corporation (http://www.lonza.com/) and maintained according to the manufacturer's recommendations.

### Sequence analysis

Viral RNA was extracted from viruses using Trizol Reagent (Invitrogen, http://www.invitrogen.com/) according to the manufacturer's protocol. RT-PCR was done using an oligonucleotide (Uni12) complementary to the conserved 3′ end of viral RNA [61]. Gene cloning and sequencing were done on at least 3 independent clones per segment as described previously [62].

The nucleotide sequence data analyzed for viruses in this study are available in the DDBJ/EMBL/GenBank databases under the accession numbers AB601121 to AB601156.

### Generation of viruses by reverse genetics

Recombinant viruses were generated with a plasmid-based reverse genetics system [63]. The viral complementary DNAs were cloned into pUC18-based plasmids, between the human RNA polymerase I promoter and the hepatitis delta virus ribozyme (pPOLI). All viruses generated by reverse genetics carried the HA gene of one of the viruses being studied, with the other genes coming from EG/D1. The HA genes of EG/0929, EG/4822 and EG/2039 were synthesized using the sequences registered in the NCBI database Influenza Virus Resource (IVR, http://www.ncbi.nlm.nih.gov/genomes/FLU/FLU.html) and site-directed mutagensis PCR (GeneTailor Site-Directed Mutagenesis System; Invitrogen). Mutant HA genes were generated by PCR-based site-directed mutagenesis in the EG/D1, EG/12, EG/0929, EG/4822 or Eg/2039 HA background. All constructs were sequenced completely to ensure the absence of unwanted mutations. Recombinant viruses were generated by plasmid transfection of co-cultured 293T and CEF cells, and were propagated in eggs. The HA genes of the virus stocks were sequenced to detect the possible emergence of revertants during amplification.

### Genetic analysis

For phylogenetic analysis of HA genes, published HA sequences of 85 representative H5N1 influenza A viruses isolated in Egypt from 2006 to 2009 were obtained from the NCBI database (http://www.ncbi.nlm.nih.gov/nucleotide). Phylogenetic analysis was performed on those 85 HA sequences and on the HA sequences of the 21 viruses isolated in this study using MEGA4 software [64] for the neighbor-joining method, with the nucleotide sequences covering most of HA gene. Estimates of the phylogenies were calculated by performing 1,000 bootstrap replicates. For phylogenetic analysis of NA genes, published NA sequences from the NCBI database of 65 representative H5N1 viruses isolated in Egypt from 2006 to 2009 together with the NA sequences of 19 viruses isolated in this study were analyzed. For a database search, published sequences of 260 HA genes from influenza A viruses isolated in Egypt from 2006 to 2009 from NCBI IVR were analyzed. For comparison, published HA sequences of 495 H5N1 influenza A viruses recently identified in Asia were also obtained from NCBI IVR. These sequences were aligned by the MAFFT program [65] and the HA1 regions were compared with the sequences of the viruses isolated in this study.

### Hemagglutination titration

Stocks of avian and human influenza viruses were serially diluted with PBS and mixed with 0.5% chicken red blood cells and 0.75% guinea pig red blood cells, respectively. Hemagglutination by avian and human influenza viruses was observed after incubation at room temperature for 30 min or 1 h, respectively to determine their HAU. To correct for differences in HAU values due to different blood lots, a reference virus sample was used and HAU values of all virus samples were adjusted relative to the reference HAU titer of EG/D1, which was used in the optimization analysis of the following receptor specificity assay.

### Receptor specificity assay

Receptor binding specificity was analyzed by a solid-phase direct binding assay as described previously [23], [24], [52], with a sialylglycopolymer containing *N*-acetylneuraminic acid linked to galactose through either an α2,3 or α2,6 bond (Neu5Acα2,3LacNAcb-pAP, and Neu5Acα2,6LacNAcb-pAP). Serial dilutions of each sialylglycopolymer were prepared in PBS, and 100 µl was added to each well of 96-well microtiter plates (Polystyrene Universal-Bind Microplates, Corning, http://www.corning.com/). The plates were then irradiated with 254 nm ultraviolet light for 10 min and each well was washed three times with 250 µl PBS. Each well was blocked with 100 µl PBS containing 0.1% Tween 20 (PBST) and 2% bovine serum albumin at room temperature for 1 h. After washing with ice-cold PBST, a solution containing influenza viruses (64 HAU in PBST) was added to each well and the plates were incubated at 4°C for 12 h. After washing five times with ice-cold PBST, mouse anti-NP antibody (against influenza virus NP protein) was added to each well and the plates were incubated at 4°C for 2 h. The wells were then washed five times with ice-cold PBST and incubated with peroxidase-conjugated goat anti-immunoglobulin (Histofine Simple Stain MAX-PO, Nichirei, http://www.nichirei.co.jp/bio/english/) at 4°C for 2 h. After washing five times with ice-cold PBST, 100 µl premixed tetrametylbenzidine-H_2_O_2_ substrate was added to each well. After incubation at room temperature for 10 min, the reactions were stopped with 50 µl 1 M H_2_SO_4_, and absorbance at 450/630 nm was measured.

Binding data were plotted against the concentration of sialic acid residues in the reaction solution and were analyzed using GraphPad Prism version 5.0 (GraphPad Software, http://www.graphpad.com/). To determine the apparent association constant (*K*a) values, nonlinear regression was used to fit the data based on the one-site model. Each data point is the mean ± SD of three to six experiments, which were each performed in triplicate.

### Viral growth kinetics in SAEC and CEF cells

SAEC cells were infected in triplicate with the indicated viruses at an MOI of 1 or 0.1. The virus inoculum was removed after 1 h and the cells were washed and airway epithelial growth medium (SAGM; Lonza) containing bovine pituitary extract (BPE; 30 µg/ml), hydrocortisone (0.5 µg/ml), human epidermal growth factor (hEGF; 0.5 ng/ml), epinephrine (0.5 µg/ml), transferrin (10 µg/ml), insulin (5 µg/ml), triiodothyronine (6.5 ng/ml), bovine serum albumin-fatty-acid free (BSA-FAF; 50 µg/ml), retinoic acid (RA; 0.1 ng/ml), gentamycin (30 µg/ml) and amphotericin B (15 ng/ml) was added. Acetylated trypsin (2 µg/ml, Sigma-Aldrich, http://www.sigmaaldrich.com/) was also added to SAEC cultures for propagation of human H3N2 virus. At the indicated times post-infection, virus titers in the cell culture supernatants were determined in triplicate by FFU assays in CEF. For determination of viral growth in CEF cells, the cells were infected in triplicate at an MOI of 0.1 or 0.01. At the indicated times post-infection, virus titers were determined in triplicate by FFU assays. Preliminary lectin-based flow cytochemistry studies indicated a difference in SA expression on the surface of SAEC and CEF cells without growth under air-liquid interface conditions, with predominant expression of α2,6 SA in SAEC cells and of α2,3 SA in CEF cells. Therefore, all cell cultures in this study were established without air-liquid interface conditions as described previously [47], [66].

### Virus histochemistry in tissue sections

To produce fluorescein isothiocyanate (FITC)-labeled viruses for histochemistry, influenza viruses, purified and concentrated as described above, were inactivated with formalin in PBS (0.025% final concentration) for 24 h at 37°C. The virus mixture was then dialyzed against PBS for 18 h at 4°C and complete inactivation was confirmed by assay on MDCK cells. A 1 ml sample of inactivated virus was then mixed with 0.1 ml 1.1 M carbonate-bicarbonate buffer (pH 9.5) containing 0.55 mg FITC isomer Ι (Invitrogen)/ml for 1 h at room temperature with constant stirring, followed by dialysis of the mixture against PBS for 42 h at 4°C. To check for hemagglutination activity by the inactivated virus, the viral hemagglutination titer was assayed after formalin inactivation and FITC labeling.

Formalin-fixed paraffin-embedded human respiratory tract tissue sections were obtained from US Biomax, Inc. (http://www.biomax.us/). The paraffin-embedded tissues were deparaffinized with xylene and hydrated using graded alcohols. After blocking with Carbo-Free Blocking Solution (Vector Laboratories, http://www.vectorlabs.com/), the tissues were then blocked with Blocking Reagent (Perkin Elmer, http://www.perkinelmer.com/). FITC-labeled influenza viruses were incubated with tissue sections at 4°C for 12 h at a titer of 128 HAU per section. The FITC label was detected with peroxidase-conjugated rabbit anti-FITC antibody (Dako, http://www.dako.com/). The signal was amplified with a tyramide signal amplification system (Perkin Elmer) according to the manufacturer's instructions. Peroxidase was visualized with 3-amino-9-ethyl-carbozole (AEC+ Substrate Chromogen, Dako), resulting in a bright red precipitate. Tissues were counterstained with hematoxylin and embedded in Aquatex (Merck Chemicals, http://www.merck-chemicals.com/). Omission of FITC-labeled virus was used as a negative control. The specificity of the virus histochemistry was verified as follows. Tissue sections, deparaffinized and hydrated as described above, were treated with *Arthrobacter ureafaciens* sialidase (100 mU/ml, Nacalai Tesque, http://www.nacalai.co.jp/) in sodium acetate buffer (100 mM, pH 5.8) for 1 h at 37°C or mock-treated before performing virus histochemistry. Micrographs were taken using a Nikon Eclipse TE2000-U Inverted Microscope (Nikon, http://www.nikon.com/).

### Experimental infections in mice

To determine MLD_50_ values, groups of 6-week-old female BALB/c mice (Japan SLC, Inc., http://www.jslc.co.jp/), under isoflurane anesthesia, were inoculated intranasally with serial 10-fold dilutions of virus in 75 µl PBS, and MLD_50_ values were calculated by the Reed-Muench method and expressed as FFU required for 1 MLD_50_. Mice were observed daily for 14 d for weight loss and mortality. Mice that lost >30% of their original weight were euthanized. At 4 and 7 d after inoculation with 3×10^4^ FFU and at 4 d after inoculation with 3×10^5^ FFU (because of mouse deaths before day 7 at this dose), virus titers in the lungs were assayed as FFU in CEF cells. Virus titers in lungs were expressed as log_10_ FFU. The lower limit of virus detection was 2 log_10_ FFU/lung. For histopathology analysis, mouse lungs collected at 7 d after inoculation with 3×10^4^ FFU were fixed in 4% buffered paraformaldehyde, embedded in paraffin, cut into 5 µm sections, stained with hematoxylin and eosin, and examined by light microscopy. Immunohistochemical staining for the H5 antigen was performed on deparaffinized sections using a monoclonal antibody (C43) specific for the nucleoprotein of influenza A virus by a two-step peroxidase method (Hisfine Mouse Stain Kit, Nichirei) with diaminobenzidine as the chromogen and hematoxylin as the counterstain. For controls, unrelated antibodies were used in place of the primary antibody.

### Homology modeling and docking

The crystal structure of the HA of influenza virus A/Vietnam/1194/04 (H5N1) (Protein Data Bank ID code 2IBX) [24] was used as a template for homology modeling of EG/D1, EG/D1_Q192H_, and EG/D_129Δ,I151T_ by the Molecular Operating Environment (MOE, http://www.chemcom.com). SA α2,3- and SA α2,6-linked analogs (PDBID code 1MQM and 1MQN) were used as the input for a docking study with the model HA structure using MOE ASEDock [67]. The MMFF94x force field and the generalized Born (GB) solvation model were used for the minimization step. The complexes were evaluated by U_dock_ scores which show the affinity between ligand and receptor. Because SA α2,3- and SA α2,6-linked analogs are a disaccharide and a trisaccharide respectively, the absolute value of their U_dock_ scores cannot be compared between the complex bound to the α2,3-linked analog and that bound to the α2,6-linked analog. However, U_dock_ scores enable the binding mode of the same analog to different HAs to be compared.

## Supporting Information

Figure S1 Phylogenetic tree of NA genes of H5N1 viruses isolated in Egypt. This tree includes published NA sequences of 63 H5N1 influenza A viruses isolated in Egypt, from the National Center for Biotechnology Information database (minimum sequence length 1,150 nt), and 19 NA sequences determined in this study (sequence length 1,350 nt). The sequences analyzed in this study are marked with a black circle. Colors are used to highlight virus strains with different hosts, isolation year and sublineage.
(TIF)

Figure S2 Optimization of viral HA titers for direct binding assays. These assays were done using 4-fold dilutions of EG/D1 and EG/12 viruses (measured as HAU), with titers ranging from 512 to 8 HAU. Direct binding of viruses to sialylglycopolymers containing either α2,3-linked (blue) or α2,6-linked (red) SA was measured. Each data point is the mean ± SD of triplicate experiments.
(TIF)

Figure S3 Effect of reverse mutations in sublineage A and BI virus HAs on receptor specificity. The reverse mutations to those in Figures 3 and 4 were introduced into the HAs of sublineage A virus EG/4822 (A) and sublineage BI virus EG/2039 (B). Direct binding to sialylglycopolymers containing either α2,3-linked (blue) or α2,6-linked (red) sialic acid was measured. Mutations are indicated by subscripts. Each data point is the mean ± SD of triplicate experiments.
(TIF)

Figure S4 Specificity of virus histochemistry. Attachment of A/Japan/434/2003 (H3N2), upper two panels, and EG/D1 virus, lower two panels, to human respiratory tract tissues. Tissue sections were treated or mock-treated with *Arthrobacter ureafaciens* sialidase before performing virus histochemistry. The panels were chosen to reflect the attachment pattern in each tissue section as much as possible.
(TIF)

Table S1 Virus binding affinity to sialylglycopolymers.
(PPT)

Table S2 Virus strains encoding HA 129Δ/I151T and Q192H mutations in avian influenza virus A virus subtypes.
(PPT)

Table S3 Properties of H5N1 influenza viruses in sublineage A.
(PPT)

Table S4 Properties of H5N1 influenza viruses in sublineage BI.
(PPT)

Table S5 Properties of H5N1 influenza viruses in sublineage BΙΙ.
(PPT)
