# Supplementary figures and images for: Acquisition of Human-Type Receptor Binding Specificity by New H5N1 Influenza Virus Sublineages during Their Emergence in Birds in Egypt
Source: PLoS Pathog. 2011 May 26;7(5):e1002068. doi: 10.1371/journal.ppat.1002068 (PMC3102706; doi:10.1371/journal.ppat.1002068)

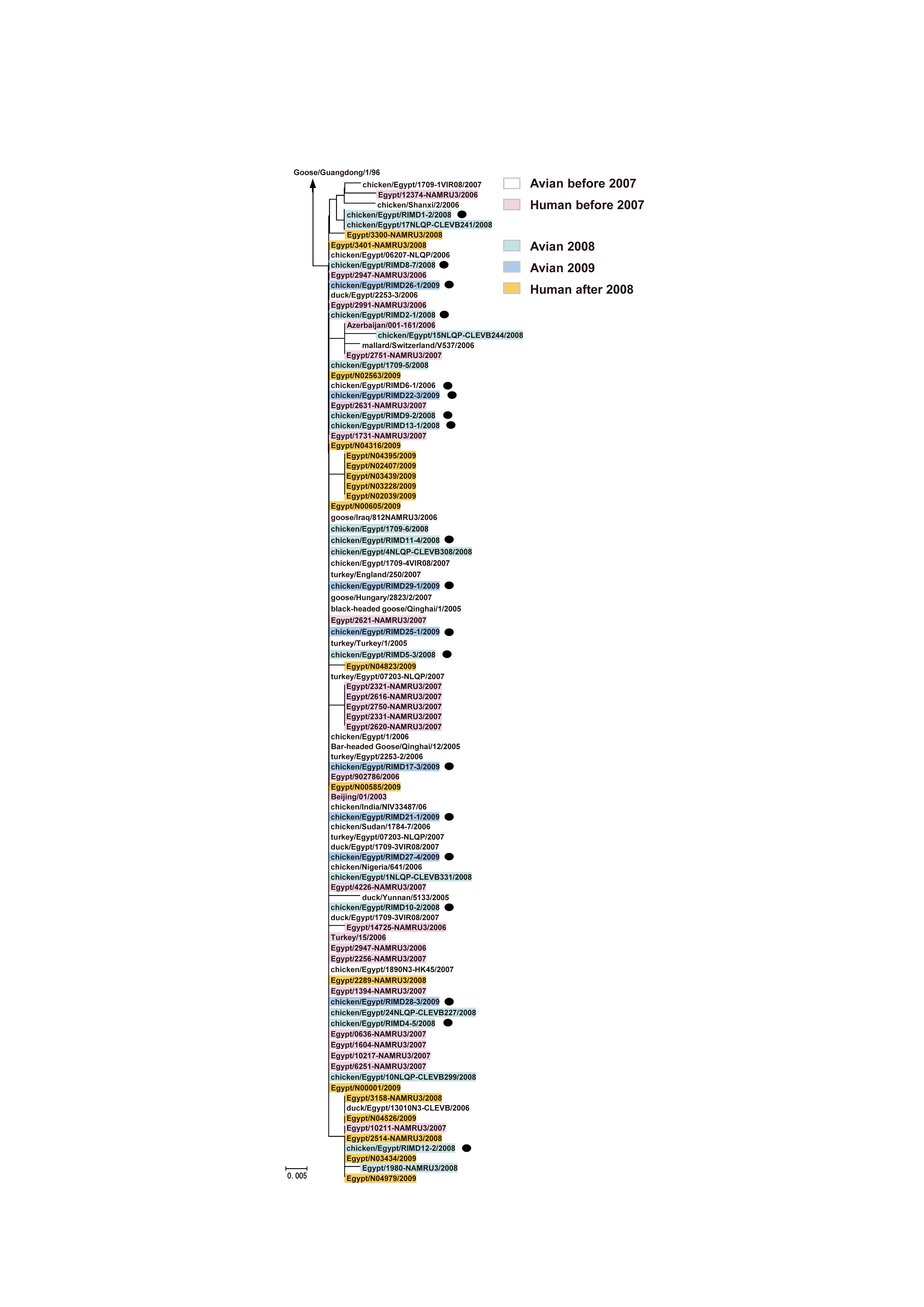

Supplement: Figure S1 — Phylogenetic tree of NA genes of H5N1 viruses isolated in Egypt. This tree includes published NA sequences of 63 H5N1 influenza A viruses isolated in Egypt, from the National Center for Biotechnology Information database (minimum sequence length 1,150 nt), and 19 NA sequences determined in this study (sequence length 1,350 nt). The sequences analyzed in this study are marked with a black circle. Colors are used to highlight virus strains with different hosts, isolation year and sublineage. (TIF) [file ppat.1002068.s001.tif]

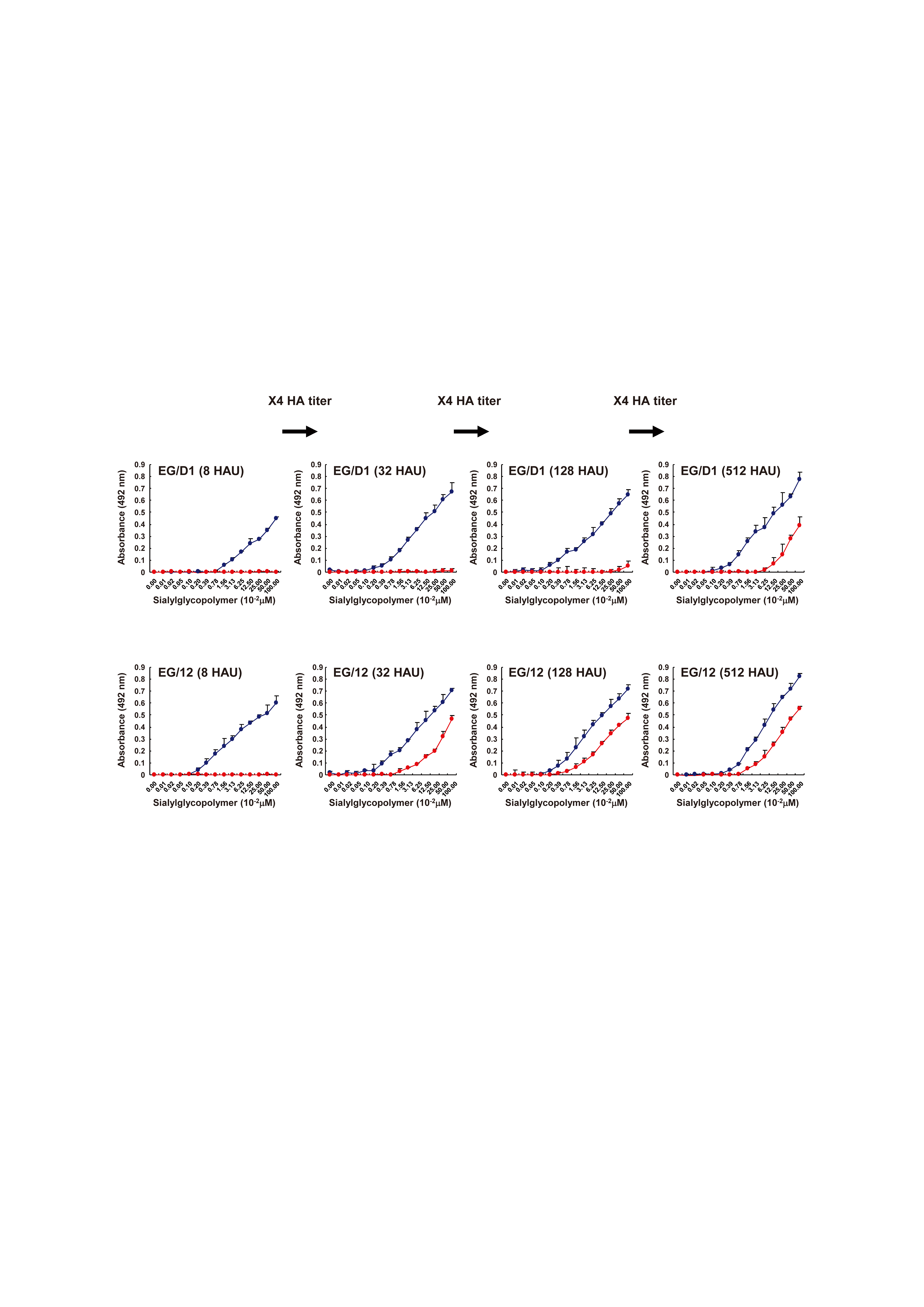

Supplement: Figure S2 — Optimization of viral HA titers for direct binding assays. These assays were done using 4-fold dilutions of EG/D1 and EG/12 viruses (measured as HAU), with titers ranging from 512 to 8 HAU. Direct binding of viruses to sialylglycopolymers containing either α2,3-linked (blue) or α2,6-linked (red) SA was measured. Each data point is the mean ± SD of triplicate experiments. (TIF) [file ppat.1002068.s002.tif]

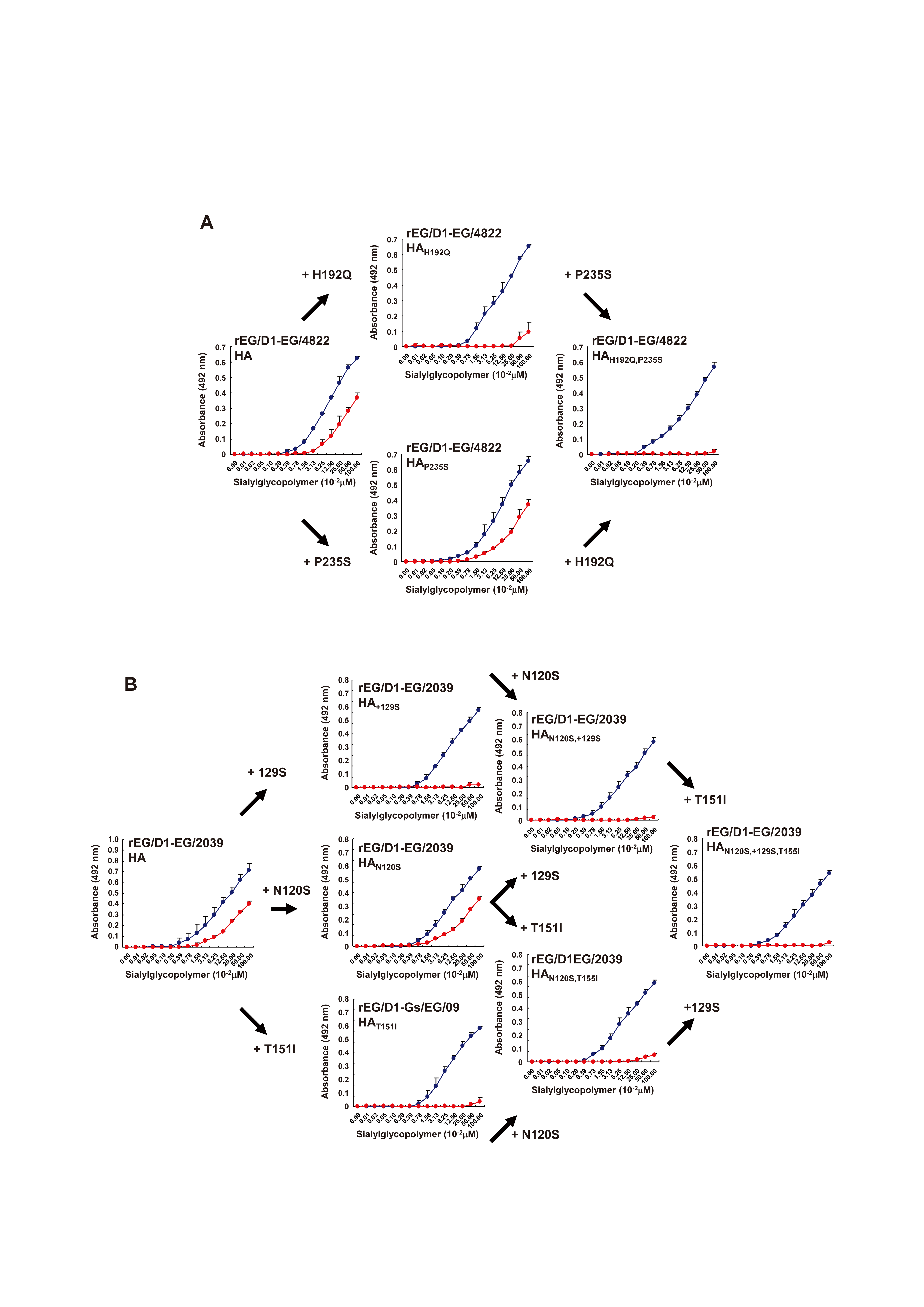

Supplement: Figure S3 — Effect of reverse mutations in sublineage A and BI virus HAs on receptor specificity. The reverse mutations to those in Figures 3 and 4 were introduced into the HAs of sublineage A virus EG/4822 (A) and sublineage BI virus EG/2039 (B). Direct binding to sialylglycopolymers containing either α2,3-linked (blue) or α2,6-linked (red) sialic acid was measured. Mutations are indicated by subscripts. Each data point is the mean ± SD of triplicate experiments. (TIF) [file ppat.1002068.s003.tif]

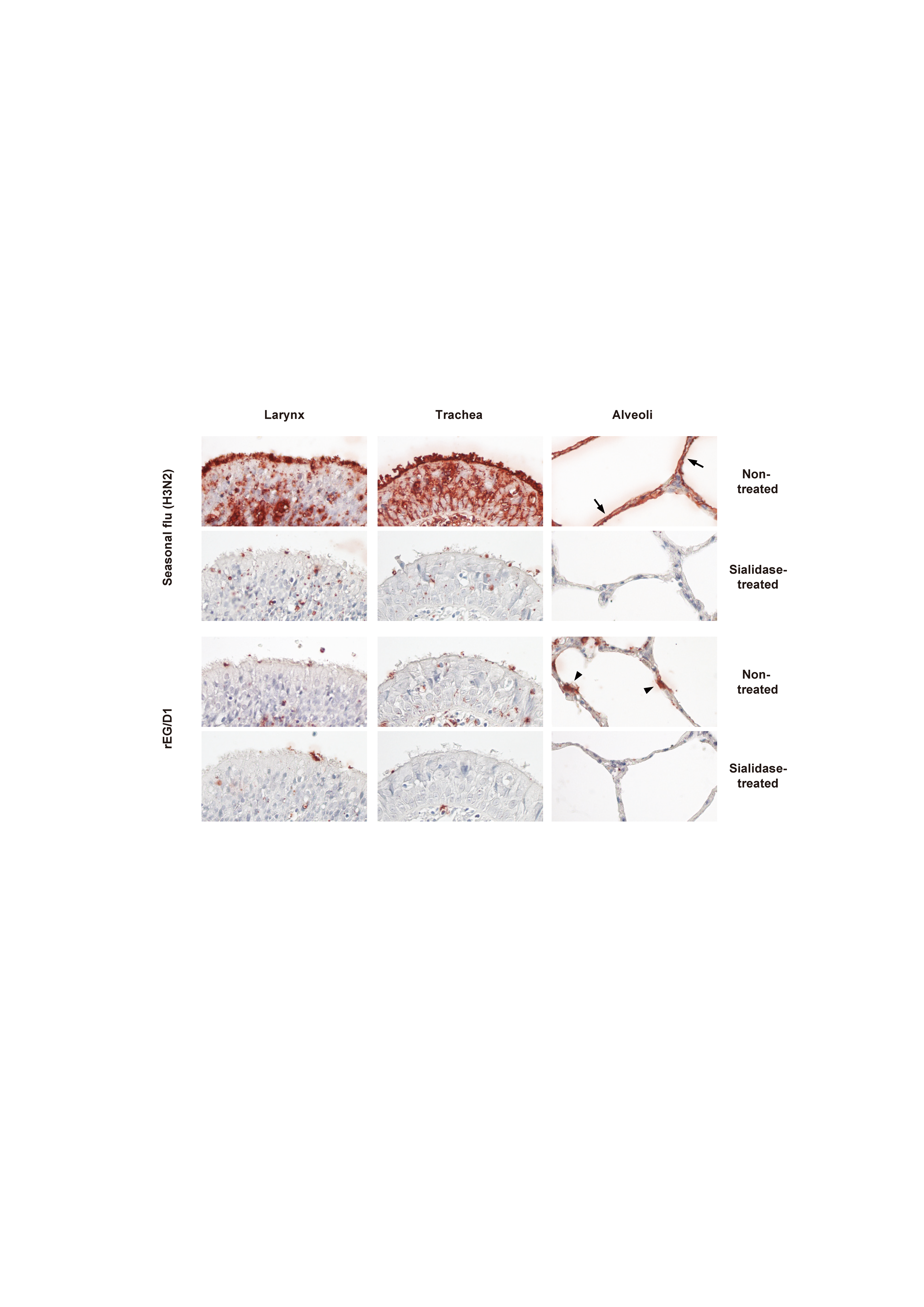

Supplement: Figure S4 — Specificity of virus histochemistry. Attachment of A/Japan/434/2003 (H3N2), upper two panels, and EG/D1 virus, lower two panels, to human respiratory tract tissues. Tissue sections were treated or mock-treated with Arthrobacter ureafaciens sialidase before performing virus histochemistry. The panels were chosen to reflect the attachment pattern in each tissue section as much as possible. (TIF) [file ppat.1002068.s004.tif]
