# Supplementary material for: Acquisition of Human-Type Receptor Binding Specificity by New H5N1 Influenza Virus Sublineages during Their Emergence in Birds in Egypt
Source: PLoS Pathog. 2011 May 26;7(5):e1002068. doi: 10.1371/journal.ppat.1002068 (PMC3102706; doi:10.1371/journal.ppat.1002068)
Supplement: Table S1 — Virus binding affinity to sialylglycopolymers. (PPT) [file ppat.1002068.s005.ppt]

## Slide 1
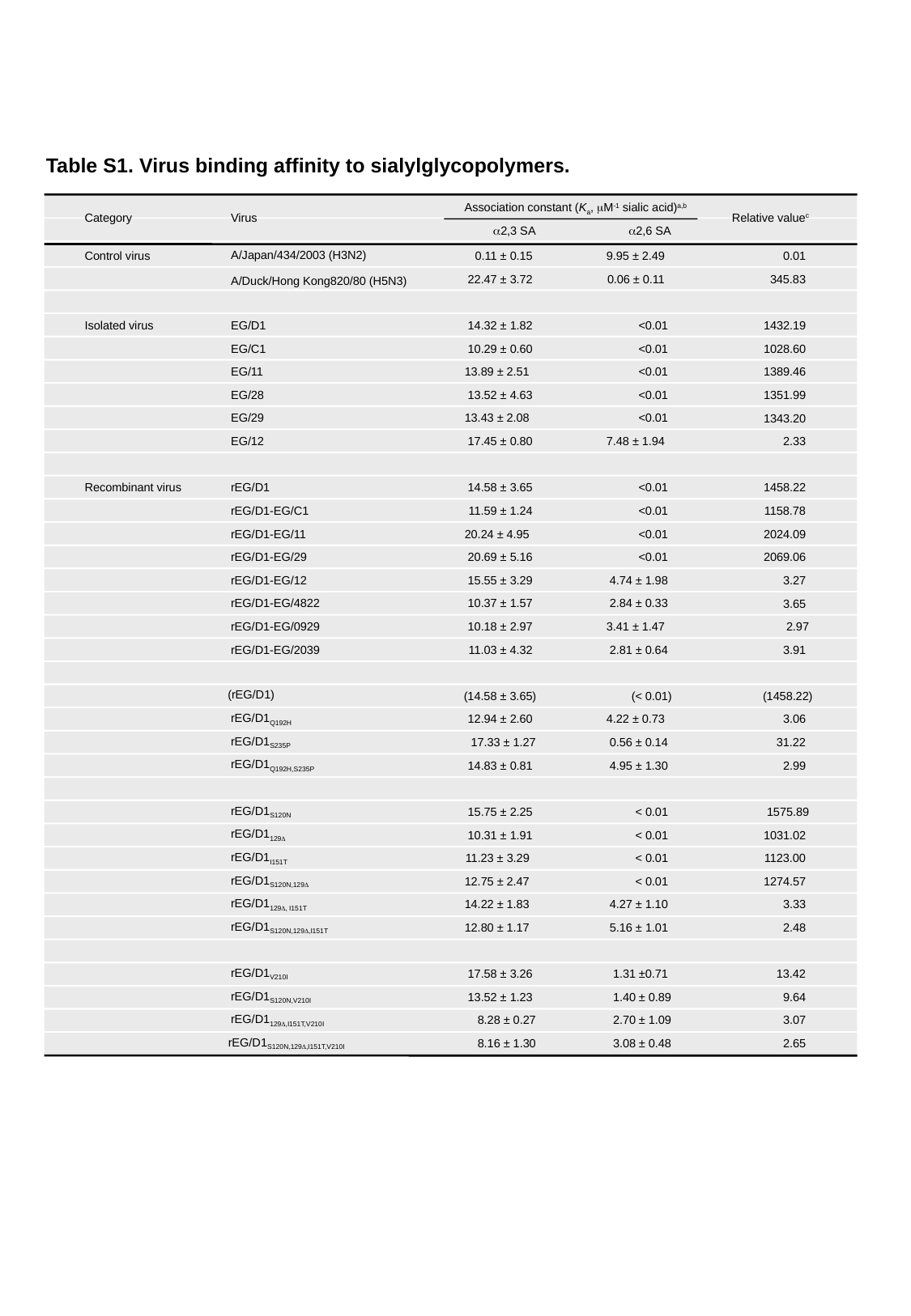

Table S1. Virus binding affinity to sialylglycopolymers.
Association constant (Ka, μM-1 sialic acid)a,b
Category
Virus
Relative valuec
α2,3 SA
α2,6 SA
Control virus
A/Japan/434/2003 (H3N2)
 0.11 ± 0.15
9.95 ± 2.49
0.01
A/Duck/Hong Kong820/80 (H5N3)
22.47 ± 3.72
0.06 ± 0.11
345.83
Isolated virus
EG/D1
14.32 ± 1.82
<0.01
1432.19
EG/C1
10.29 ± 0.60
<0.01
1028.60
EG/11
13.89 ± 2.51
<0.01
1389.46
EG/28
13.52 ± 4.63
<0.01
1351.99
EG/29
13.43 ± 2.08
<0.01
1343.20
EG/12
17.45 ± 0.80
7.48 ± 1.94
2.33
Recombinant virus
rEG/D1
14.58 ± 3.65
<0.01
1458.22
rEG/D1-EG/C1
11.59 ± 1.24
<0.01
1158.78
rEG/D1-EG/11
20.24 ± 4.95
<0.01
2024.09
rEG/D1-EG/29
20.69 ± 5.16
<0.01
2069.06
rEG/D1-EG/12
15.55 ± 3.29
4.74 ± 1.98
3.27
rEG/D1-EG/4822
10.37 ± 1.57
2.84 ± 0.33
3.65
rEG/D1-EG/0929
10.18 ± 2.97
3.41 ± 1.47
2.97
rEG/D1-EG/2039
11.03 ± 4.32
2.81 ± 0.64
3.91
(rEG/D1)
(14.58 ± 3.65)
(< 0.01)
(1458.22)
rEG/D1Q192H
12.94 ± 2.60
4.22 ± 0.73
3.06
rEG/D1S235P
17.33 ± 1.27
0.56 ± 0.14
31.22
rEG/D1Q192H,S235P
14.83 ± 0.81
4.95 ± 1.30
2.99
rEG/D1S120N
15.75 ± 2.25
< 0.01
1575.89
rEG/D1129Δ
10.31 ± 1.91
< 0.01
1031.02
rEG/D1I151T
11.23 ± 3.29
< 0.01
1123.00
rEG/D1S120N,129Δ
12.75 ± 2.47
< 0.01
1274.57
rEG/D1129Δ, I151T
14.22 ± 1.83
4.27 ± 1.10
3.33
rEG/D1S120N,129Δ,I151T
12.80 ± 1.17
5.16 ± 1.01
2.48
rEG/D1V210I
17.58 ± 3.26
1.31 ±0.71
13.42
rEG/D1S120N,V210I
13.52 ± 1.23
1.40 ± 0.89
9.64
rEG/D1129Δ,I151T,V210I
8.28 ± 0.27
2.70 ± 1.09
3.07
rEG/D1S120N,129Δ,I151T,V210I
8.16 ± 1.30
3.08 ± 0.48
2.65

## Slide 2
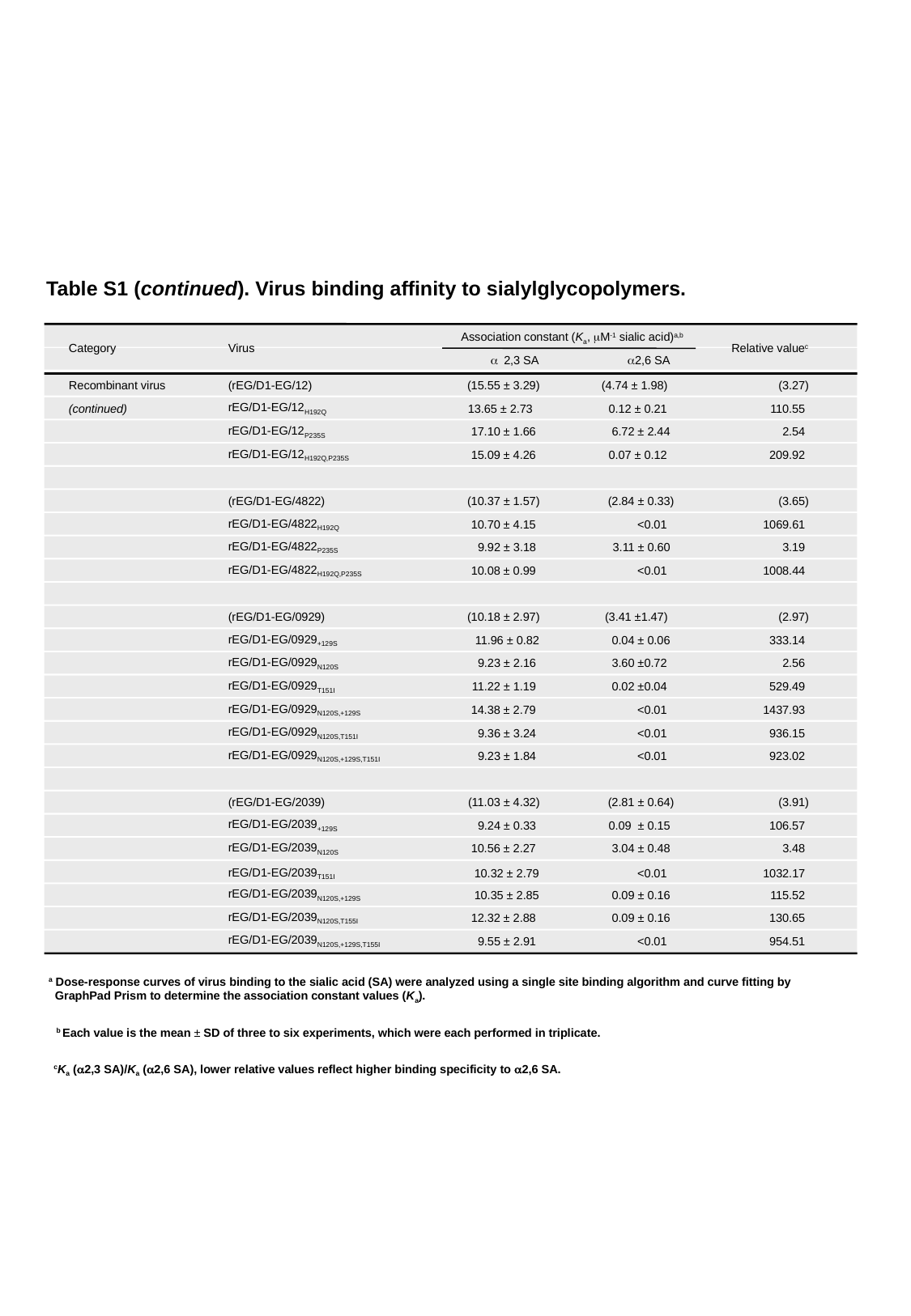

Table S1 (continued). Virus binding affinity to sialylglycopolymers.
Association constant (Ka, μM-1 sialic acid)a,b
Category
Virus
Relative valuec
α 2,3 SA
α2,6 SA
Recombinant virus
(rEG/D1-EG/12)
(15.55 ± 3.29)
(4.74 ± 1.98)
(3.27)
(continued)
rEG/D1-EG/12H192Q
13.65 ± 2.73
0.12 ± 0.21
110.55
rEG/D1-EG/12P235S
17.10 ± 1.66
6.72 ± 2.44
2.54
rEG/D1-EG/12H192Q,P235S
15.09 ± 4.26
0.07 ± 0.12
209.92
(rEG/D1-EG/4822)
(10.37 ± 1.57)
(2.84 ± 0.33)
(3.65)
rEG/D1-EG/4822H192Q
10.70 ± 4.15
<0.01
1069.61
rEG/D1-EG/4822P235S
9.92 ± 3.18
3.11 ± 0.60
3.19
rEG/D1-EG/4822H192Q,P235S
10.08 ± 0.99
<0.01
1008.44
(rEG/D1-EG/0929)
(10.18 ± 2.97)
(3.41 ±1.47)
(2.97)
rEG/D1-EG/0929+129S
11.96 ± 0.82
0.04 ± 0.06
333.14
rEG/D1-EG/0929N120S
9.23 ± 2.16
3.60 ±0.72
2.56
rEG/D1-EG/0929T151I
11.22 ± 1.19
0.02 ±0.04
529.49
rEG/D1-EG/0929N120S,+129S
14.38 ± 2.79
<0.01
1437.93
rEG/D1-EG/0929N120S,T151I
9.36 ± 3.24
<0.01
936.15
rEG/D1-EG/0929N120S,+129S,T151I
9.23 ± 1.84
<0.01
923.02
(rEG/D1-EG/2039)
(11.03 ± 4.32)
(2.81 ± 0.64)
(3.91)
rEG/D1-EG/2039+129S
9.24 ± 0.33
0.09 ± 0.15
106.57
rEG/D1-EG/2039N120S
10.56 ± 2.27
3.04 ± 0.48
3.48
rEG/D1-EG/2039T151I
10.32 ± 2.79
<0.01
1032.17
rEG/D1-EG/2039N120S,+129S
10.35 ± 2.85
0.09 ± 0.16
115.52
rEG/D1-EG/2039N120S,T155I
12.32 ± 2.88
0.09 ± 0.16
130.65
rEG/D1-EG/2039N120S,+129S,T155I
9.55 ± 2.91
<0.01
954.51
a Dose-response curves of virus binding to the sialic acid (SA) were analyzed using a single site binding algorithm and curve fitting by
 GraphPad Prism to determine the association constant values (Ka).
b Each value is the mean ± SD of three to six experiments, which were each performed in triplicate.
cKa (α2,3 SA)/Ka (α2,6 SA), lower relative values reflect higher binding specificity to α2,6 SA.
