# Supplementary material for: Acquisition of Human-Type Receptor Binding Specificity by New H5N1 Influenza Virus Sublineages during Their Emergence in Birds in Egypt
Source: PLoS Pathog. 2011 May 26;7(5):e1002068. doi: 10.1371/journal.ppat.1002068 (PMC3102706; doi:10.1371/journal.ppat.1002068)
Supplement: Table S2 — Virus strains encoding HA 129Δ/I151T and Q192H mutations in avian influenza virus A virus subtypes. (PPT) [file ppat.1002068.s006.ppt]

## Slide 1
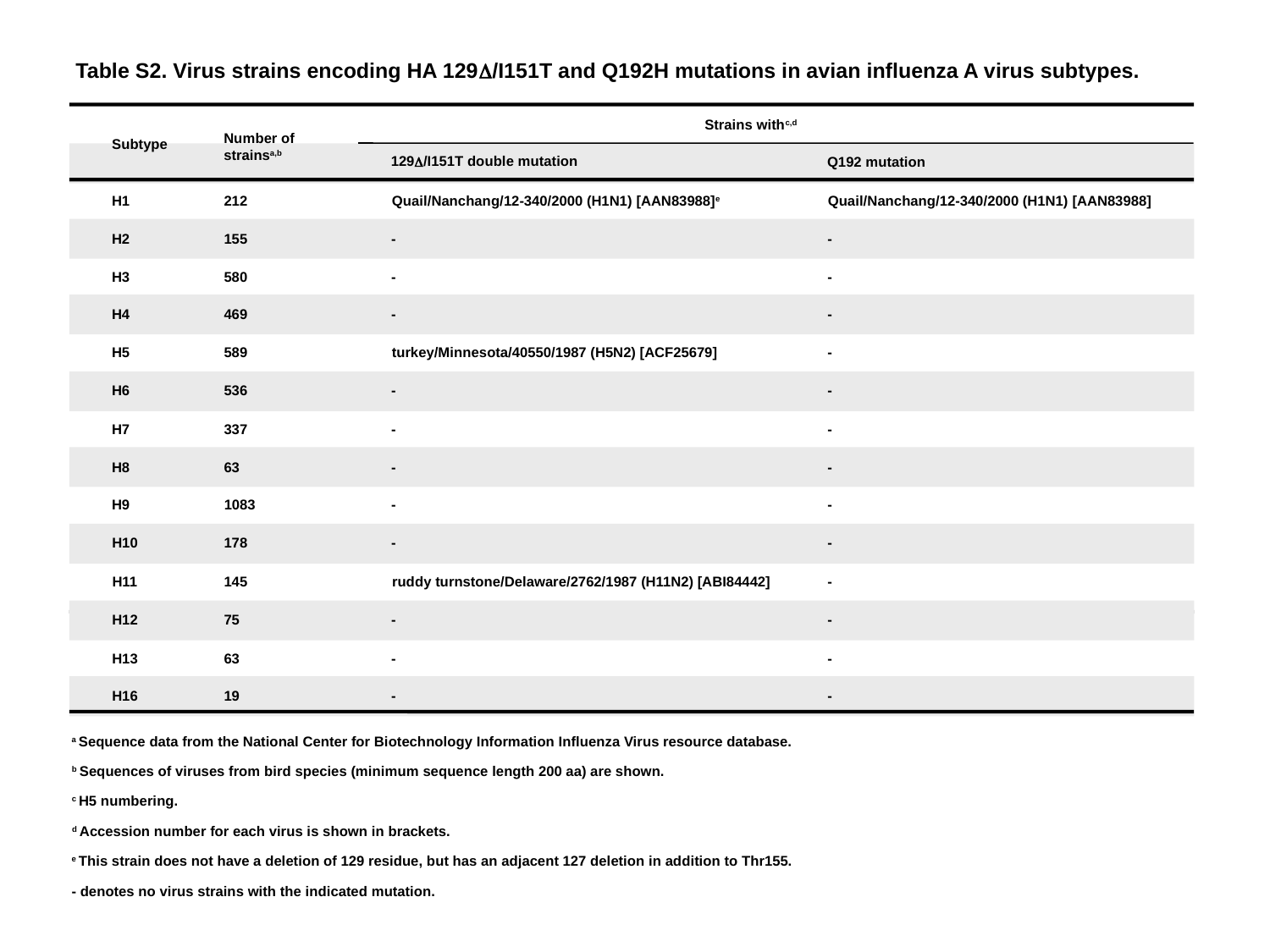

Table S2. Virus strains encoding HA 129Δ/I151T and Q192H mutations in avian influenza A virus subtypes.
Strains withc,d
Number of
strainsa,b
Subtype
129Δ/I151T double mutation
Q192 mutation
H1
H2
H3
H4
H5
H6
H7
H8
H9
H10
H11
H12
H13
H16
212
Quail/Nanchang/12-340/2000 (H1N1) [AAN83988]e
Quail/Nanchang/12-340/2000 (H1N1) [AAN83988]
155
-
-
580
-
-
469
-
-
589
turkey/Minnesota/40550/1987 (H5N2) [ACF25679]
-
536
-
-
337
-
-
63
-
-
1083
-
-
178
-
-
145
ruddy turnstone/Delaware/2762/1987 (H11N2) [ABI84442]
-
75
-
-
63
-
-
19
-
-
a Sequence data from the National Center for Biotechnology Information Influenza Virus resource database.
b Sequences of viruses from bird species (minimum sequence length 200 aa) are shown.
c H5 numbering.
d Accession number for each virus is shown in brackets.
e This strain does not have a deletion of 129 residue, but has an adjacent 127 deletion in addition to Thr155.
- denotes no virus strains with the indicated mutation.
