# Supplementary material for: Acquisition of Human-Type Receptor Binding Specificity by New H5N1 Influenza Virus Sublineages during Their Emergence in Birds in Egypt
Source: PLoS Pathog. 2011 May 26;7(5):e1002068. doi: 10.1371/journal.ppat.1002068 (PMC3102706; doi:10.1371/journal.ppat.1002068)
Supplement: Table S3 — Properties of H5N1 influenza viruses in sublineage A. (PPT) [file ppat.1002068.s007.ppt]

## Slide 1
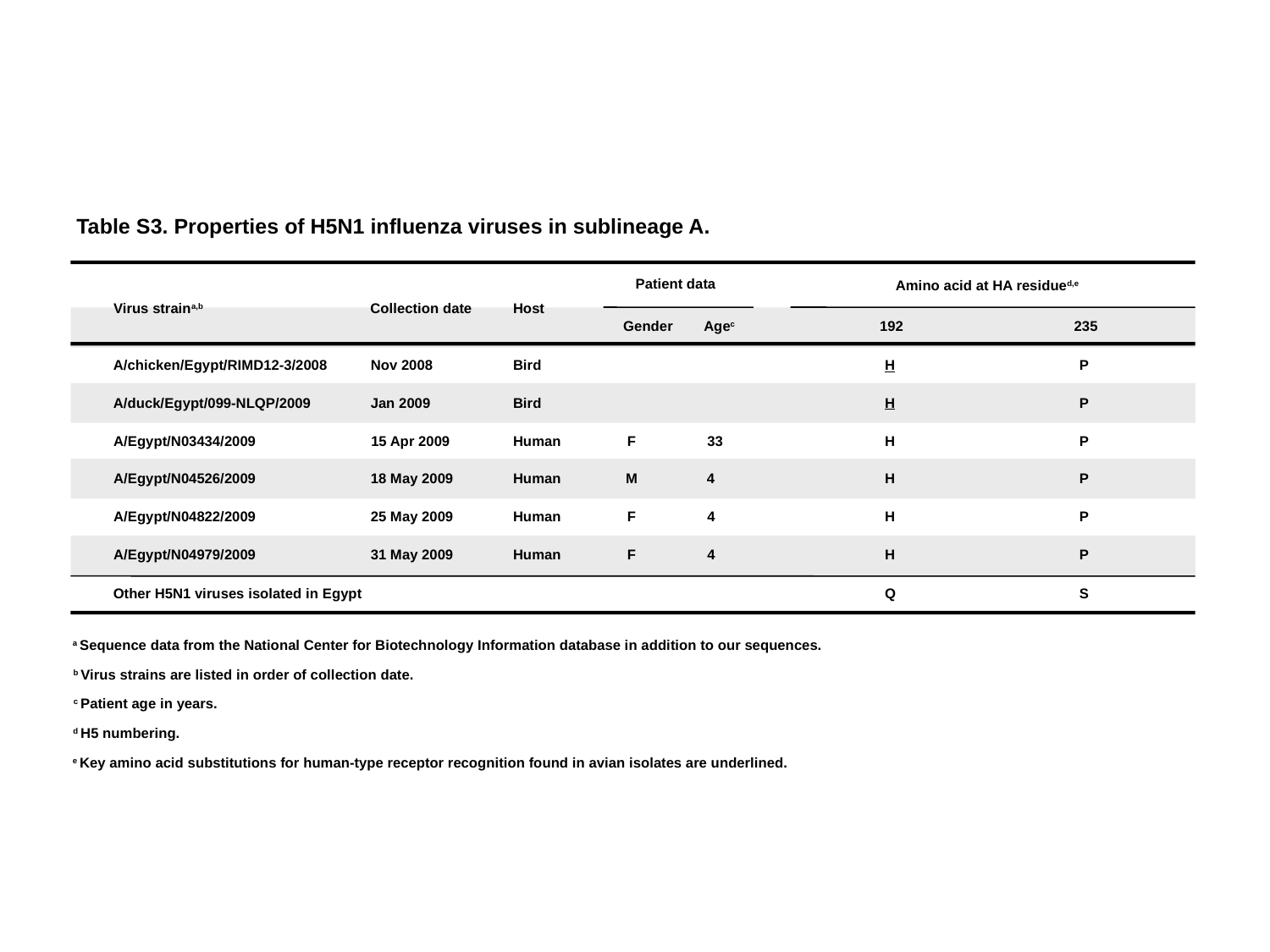

Table S3. Properties of H5N1 influenza viruses in sublineage A.
Patient data
Amino acid at HA residued,e
Virus straina,b
Collection date
Host
Gender
Agec
192
235
A/chicken/Egypt/RIMD12-3/2008
Nov 2008
Bird
H
P
A/duck/Egypt/099-NLQP/2009
Jan 2009
Bird
H
P
A/Egypt/N03434/2009
15 Apr 2009
Human
F
33
H
P
A/Egypt/N04526/2009
18 May 2009
Human
M
4
H
P
A/Egypt/N04822/2009
25 May 2009
Human
F
4
H
P
A/Egypt/N04979/2009
31 May 2009
Human
F
4
H
P
Other H5N1 viruses isolated in Egypt
Q
S
a Sequence data from the National Center for Biotechnology Information database in addition to our sequences.
b Virus strains are listed in order of collection date.
c Patient age in years.
d H5 numbering.
e Key amino acid substitutions for human-type receptor recognition found in avian isolates are underlined.
