# Supplementary material for: Acquisition of Human-Type Receptor Binding Specificity by New H5N1 Influenza Virus Sublineages during Their Emergence in Birds in Egypt
Source: PLoS Pathog. 2011 May 26;7(5):e1002068. doi: 10.1371/journal.ppat.1002068 (PMC3102706; doi:10.1371/journal.ppat.1002068)
Supplement: Table S4 — Properties of H5N1 influenza viruses in sublineage BI. (PPT) [file ppat.1002068.s008.ppt]

## Slide 1
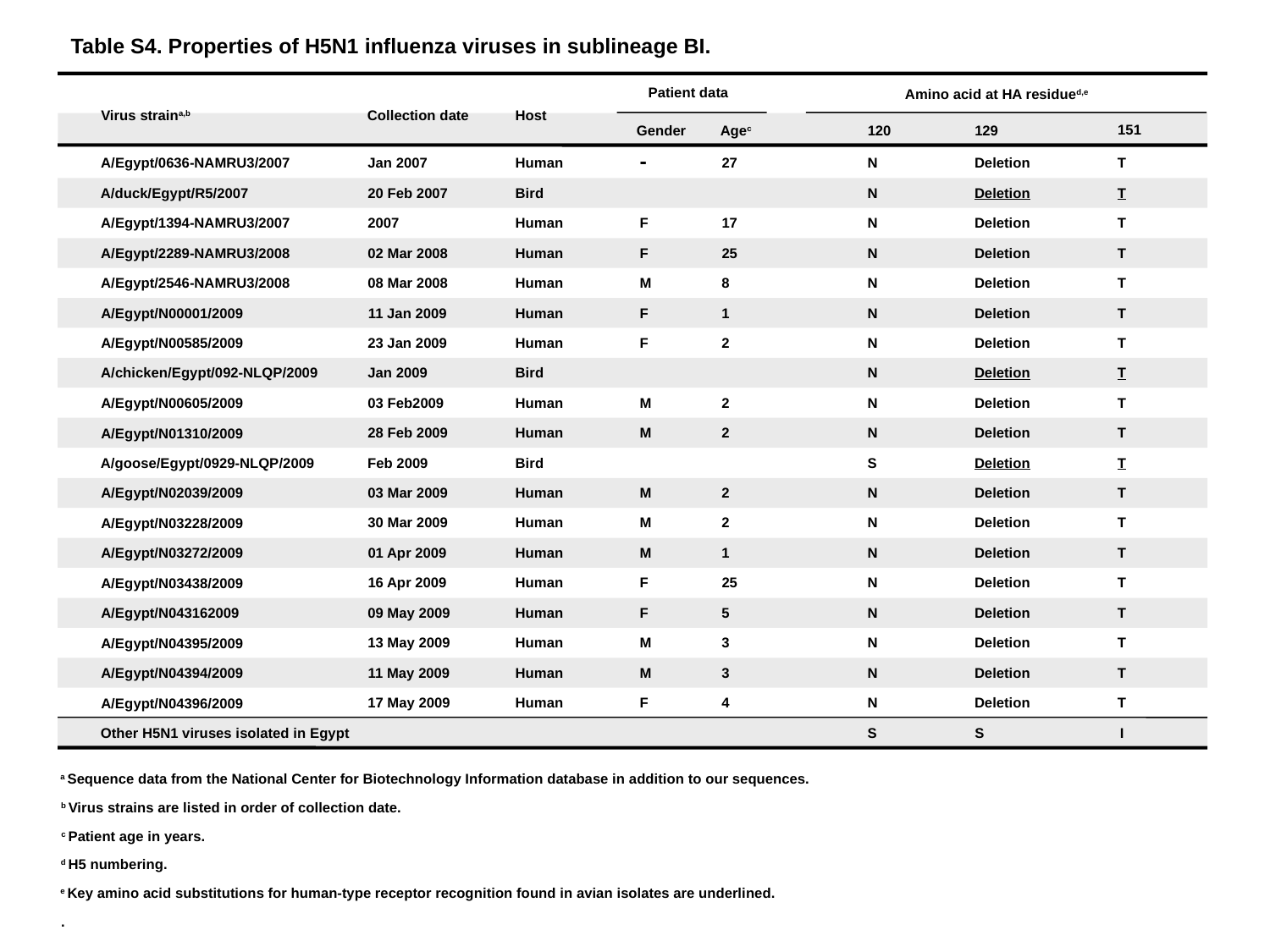

Table S4. Properties of H5N1 influenza viruses in sublineage BI.
Patient data
Amino acid at HA residued,e
Virus straina,b
Collection date
Host
151
Gender
Agec
120
129
-
A/Egypt/0636-NAMRU3/2007
Jan 2007
Human
27
N
Deletion
T
A/duck/Egypt/R5/2007
20 Feb 2007
Bird
N
Deletion
T
A/Egypt/1394-NAMRU3/2007
2007
Human
F
17
N
Deletion
T
A/Egypt/2289-NAMRU3/2008
02 Mar 2008
Human
F
25
N
Deletion
T
A/Egypt/2546-NAMRU3/2008
08 Mar 2008
Human
M
8
N
Deletion
T
A/Egypt/N00001/2009
11 Jan 2009
Human
F
1
N
Deletion
T
A/Egypt/N00585/2009
23 Jan 2009
Human
F
2
N
Deletion
T
A/chicken/Egypt/092-NLQP/2009
Jan 2009
Bird
N
Deletion
T
A/Egypt/N00605/2009
03 Feb2009
Human
M
2
N
Deletion
T
28 Feb 2009
Human
M
2
N
Deletion
T
A/Egypt/N01310/2009
Feb 2009
Bird
S
Deletion
T
A/goose/Egypt/0929-NLQP/2009
03 Mar 2009
Human
M
2
N
Deletion
T
A/Egypt/N02039/2009
30 Mar 2009
Human
M
2
N
Deletion
T
A/Egypt/N03228/2009
01 Apr 2009
Human
M
1
N
Deletion
T
A/Egypt/N03272/2009
16 Apr 2009
Human
F
25
N
Deletion
T
A/Egypt/N03438/2009
09 May 2009
Human
F
5
N
Deletion
T
A/Egypt/N043162009
13 May 2009
Human
M
3
N
Deletion
T
A/Egypt/N04395/2009
11 May 2009
Human
M
3
N
Deletion
T
A/Egypt/N04394/2009
17 May 2009
Human
F
4
N
Deletion
T
A/Egypt/N04396/2009
Other H5N1 viruses isolated in Egypt
S
S
I
a Sequence data from the National Center for Biotechnology Information database in addition to our sequences.
b Virus strains are listed in order of collection date.
c Patient age in years.
d H5 numbering.
e Key amino acid substitutions for human-type receptor recognition found in avian isolates are underlined.
.
