# Supplementary material for: Acquisition of Human-Type Receptor Binding Specificity by New H5N1 Influenza Virus Sublineages during Their Emergence in Birds in Egypt
Source: PLoS Pathog. 2011 May 26;7(5):e1002068. doi: 10.1371/journal.ppat.1002068 (PMC3102706; doi:10.1371/journal.ppat.1002068)
Supplement: Table S5 — Properties of H5N1 influenza viruses in sublineage BΙΙ. (PPT) [file ppat.1002068.s009.ppt]

## Slide 1
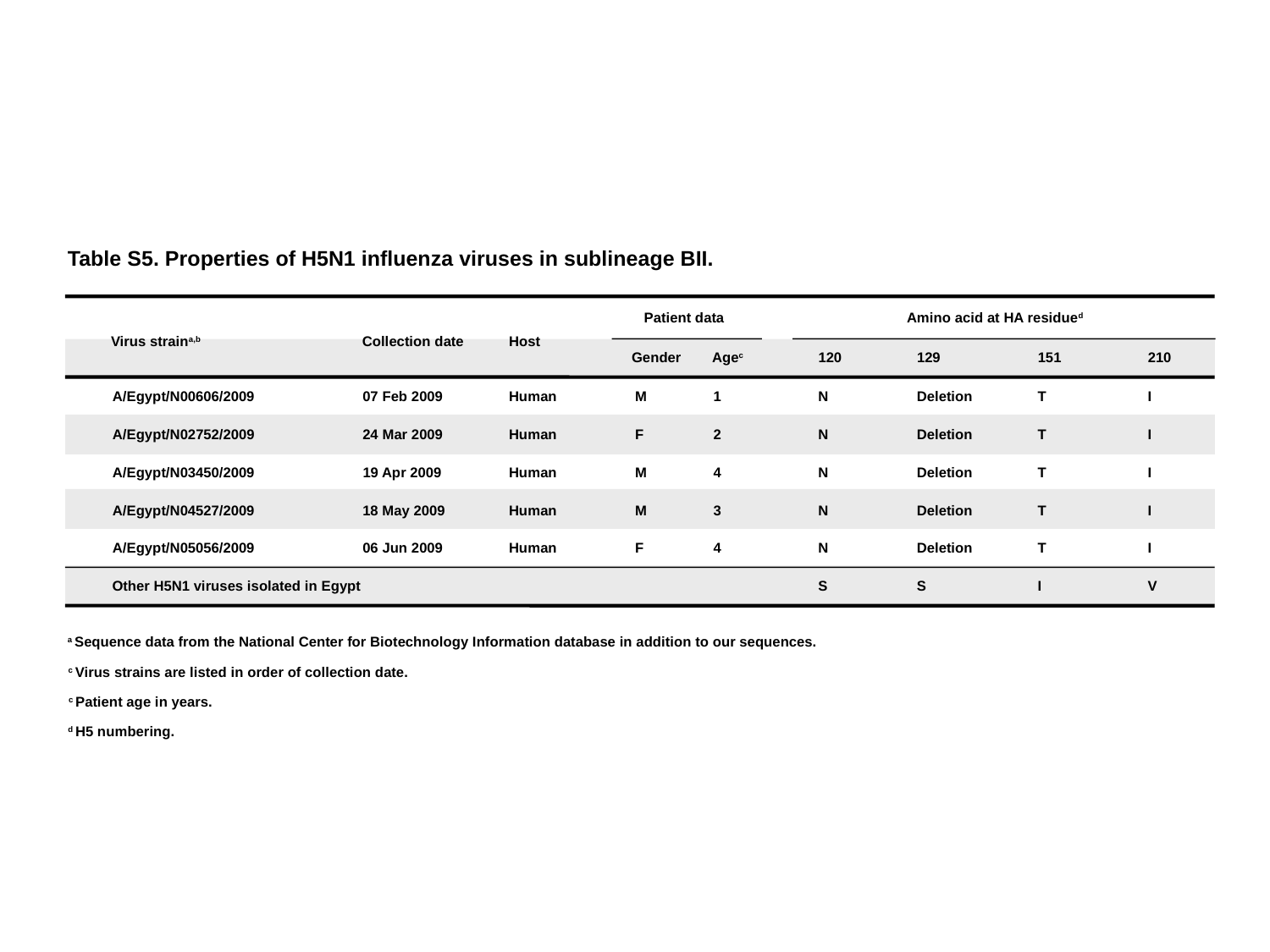

Table S5. Properties of H5N1 influenza viruses in sublineage BII.
Patient data
Amino acid at HA residued
Virus straina,b
Collection date
Host
Gender
Agec
120
129
151
210
A/Egypt/N00606/2009
07 Feb 2009
Human
M
1
N
Deletion
T
I
A/Egypt/N02752/2009
24 Mar 2009
Human
F
2
N
Deletion
T
I
A/Egypt/N03450/2009
19 Apr 2009
Human
M
4
N
Deletion
T
I
A/Egypt/N04527/2009
18 May 2009
Human
M
3
N
Deletion
T
I
A/Egypt/N05056/2009
06 Jun 2009
Human
F
4
N
Deletion
T
I
Other H5N1 viruses isolated in Egypt
S
S
I
V
a Sequence data from the National Center for Biotechnology Information database in addition to our sequences.
c Virus strains are listed in order of collection date.
c Patient age in years.
d H5 numbering.
